# Endophytic *Bacillus* Bacteria Living in Sugarcane Plant Tissues and *Telchin licus licus* Larvae (Drury) (Lepidoptera: Castniidae): The Symbiosis That May Open New Paths in the Biological Control

**DOI:** 10.3389/fmicb.2021.659965

**Published:** 2021-05-12

**Authors:** Francine Yuriko Otsuka Rocha, Aldomário Santo Negrisoli Júnior, Gustavo Feitosa de Matos, Patrícia de Medeiros Gitahy, Carolina Nachi Rossi, Marcia Soares Vidal, José Ivo Baldani

**Affiliations:** ^1^Crop Science Graduate Course, Agronomy Institute, Federal Rural University of Rio de Janeiro, Seropédica, Brazil; ^2^Laboratory of Genetics and Biochemistry, Embrapa Agrobiologia, Seropédica, Brazil; ^3^Embrapa Tabuleiros Costeiros (UEP-Rio Largo, Campus Delza Gitai), Federal University of Alagoas, Maceió, Brazil; ^4^Laboratory of Microbial Ecology, Embrapa Agrobiologia, Seropédica, Brazil

**Keywords:** insect–microbe interaction, giant borer, diversity, taxonomy, ecological niche

## Abstract

Bacteria of the genus *Bacillus* can colonize endophytically and benefit several crops including the control of some pest orders. In view of the benefits provided by these microorganisms and in order to find out an efficient biotechnological control for the giant borer, our interest in studying the microorganisms in symbiosis with sugarcane and the giant borer has arisen, since there is no efficient chemical or biological control method for this pest. Therefore, endophytic *Bacillus* strains were isolated from three sugarcane niches (apoplast fluid, central internode cylinder and roots) and also from the giant borer larvae living inside sugarcane varieties grown in the Northeast region of Brazil. The taxonomical characterization (16S rRNA) of 157 Gram-positive isolates showed that 138 strains belonged to the *Bacillus* genus. The most representative species were phylogenetically closely related to *B. megaterium* (11.5%) followed by *B. safensis* (10.8%), *B. cereus* (8.9%), *B. oleronius* (8.9%), *B. amyloliquefaciens* (7.0%), and *B. pacificus* (6.4%). BOX-PCR analyses showed very distinct band pattern profiles suggesting a great diversity of *Bacillus* species within the sugarcane niches and the digestive tract, while the *B. cereus* group remained very closely clustered in the dendrogram. According to XRE biomarker analysis, eleven strains (FORCN005, 007, 008, 011, 012, 014, 067, 076, 092, 093, and 135) correspond to *B. thuringiensis* species. Additional studies using conserved genes (*glp*, *gmk*, *pta*, and *tpi*) indicated that most of these strains were phylogenetically closely related to *B. thuringiensis* and may be considered different subspecies. In conclusion, this study suggests that the culturable *Bacillus* species are greatly diversified within the plant niches and showed *Bacillus* species in the digestive tract of the giant borer for the first time. These results open new perspectives to understand the role and functions played by these microorganisms in symbiosis with this pest and also the possibility of developing an efficient biological control method for the giant borer using strains identified as the *B. thuringiensis* species.

## Introduction

Endophytic bacteria are known to colonize, at some stage of their life cycle, internal tissues of the host plants without causing any visual pathogenic symptoms in plant cells (Azevedo et al., 2000; Hardoim et al., 2015). Endophytic microorganisms can promote host plant development, as has been shown in sugarcane plants inoculated with endophytic bacteria, which can produce auxin, secrete siderophore and solubilize phosphorus (Wang et al., 2019). In addition, endophytic microorganisms have also shown antagonistic activity against several pathogenic bacteria and fungi (Lanna-Filho et al., 2017; Shahzad et al., 2017; Wang et al., 2019).

Another important contribution of endophytic bacteria is their promising entomopathogenic potential, as has been demonstrated for the control of economically important pests (Bensidhoum et al., 2016; Schellenberger et al., 2016). Among the *Bacillus* species, the most used in biological pest control is the bacterium *Bacillus thuringiensis* (Bravo et al., 2017), which accounts for 53% of the biopesticides market (Polanczyk et al., 2017). However, despite the great success of using *B. thuringiensis*, there are some disadvantages involved in the application of the available formulations (Sanahuja et al., 2011).

One alternative that should be considered is the use of endophytic *Bacillus* bacteria to control pests that attack the main crops. Once inside the plant tissues, the endophytic microorganisms are not affected by external environmental conditions such as temperature, salinity, ultraviolet radiation, osmotic potential, pH and drought stress (Suman et al., 2016). For example, Melatti et al. (2010) demonstrated the high toxicity of five *Bacillus thuringiensis* strains against *Aphis gossypii* (Hemiptera: Aphididae), with two strains causing mortality rates of around 70%. Monnerat et al. (2003) also observed that larvae of *Spodoptera frugiperda* (Lepidoptera: Noctuidae) died 5 days after being fed with cotton leaves inoculated with *B. thuringiensis*. In another study, the same authors observed the ability of *B. thuringiensis* strain HD-1 to endophytically colonize cotton and cabbage plants and, in addition, to cause toxicity against the pests *S. frugiperda* (cotton) and *Plutella xylostella* (Lepidoptera: Plutellidae) (cabbage) (Monnerat et al., 2009). This work opened new avenues for using *Bacillus* species as an endophyte, acting in the control of different pests. It is believed that its endophytic behavior makes this microorganism a very promising alternative since it can remain in cultivated areas for longer, enhancing the pest control and, with that, the development of the plant. Therefore, these characteristics turn the endophytic *Bacillus* into potential agents for the biological control of target pests (Lodewyckx et al., 2002).

Knowledge about the *Bacillus* bacterial species which live endophytically in different tissues associated with the bioinsecticide functions is the initial step in searching for bacteria involved in plant growth development and in the control of pests and diseases (Paniagua Voirol et al., 2018). One of the major pests which attacks sugarcane crop grown in the Northeast region of Brazil is the giant borer (*Telchin licus licus*) (Lepidoptera: Castniidae) and it causes damage equivalent to 65% of yield (Linares et al., 1996). There is neither an efficient chemical pesticide nor a biological control agent that can minimize the damage caused by the giant borer (Negrisoli Junior et al., 2015). In this way, searching for *Bacillus* bacteria that endophytically colonize sugarcane grown in the region could allow the identification of strains with high biological potential to control the giant borer. There have been a few reports on the occurrence of *Bacillus* species associated with roots of sugarcane grown in China (Wang et al., 2019) and culm apoplast fluid of the plant grown in Cuba (Velázquez et al., 2008). Several *Bacillus* species were isolated from the apoplast fluid of sugarcane grown in Brazil (Melo et al., 2021). Besides the well-known entomopathogenic activity of *B. thuringiensis* strains on different pests, it was reported more recently that an endophytic *Brevibacillus laterosporus* (*Bl*) strain *Bl* 1951 presented an insecticidal toxic effect on the diamondback moth that attacks brassica species (Ormskirk et al., 2019). This opened new perspectives to find species of *Bacillus* with a broad range of biopesticide and biofertilizer activities in association with sugarcane with the aim of controlling the pests that attack this crop.

In this sense, this study aimed to isolate and taxonomically identify *Bacillus* strains that endophytically colonize different sugarcane niches, including the digestive tract of *T. licus licus* (giant borer), a pest that causes damage inside the stem of sugarcane plants. By identifying these microorganisms, we aim to understand their role in sugarcane plants and in the digestive tract of the giant borer, in order to contribute to future investigations into the biological control of this pest.

## Materials and Methods

### Harvest of Sugarcane Stem and Borer Larvae in Sugarcane Producing Areas

The sugarcane material used for the isolation of endophytic bacteria was collected (June 2016) from three sugarcane mills (young and healthy plant material – winter planting) in the Coruripe, Boca da Mata and São Luís do Quitunde county, Alagoas State, Northeast Brazil (Supplementary Table 1). Fifteen healthy sugarcane stems were collected per plant (five stems from a subarea of the mill) from the varieties RB92579, RB867515, RB951541 and “Pé-de-Ferro.”

A total of six internodes were used from each subarea of the mill to isolate endophytic bacteria from the apoplast fluid. After the fluid extraction, the central cylinder region of the internodes was separated for isolation of the endophytic bacteria present in the symplast tissues. The sugarcane roots collected at the three sugarcane mills were used to isolate bacilli endophytic bacteria after root surface disinfestation, as described below.

The larvae were collected at the same time from the sugarcane field at the Boca da Mata (VAT90212 and RB867515 varieties) and São Luís do Quitunde counties (RB92579 variety) and used to isolate the bacilli endophytic bacteria. For that, the digestive tracts of larvae from the Triunfo mill (Boca da Mata) were removed and divided into four subsamples (10 digestive tracts per subsample) totaling 40 larvae, while a sample containing ten digestive tracts was collected at the Santo Antônio mill (São Luís do Quitunde).

### Isolation of Bacilli Endophytic Bacteria From Sugarcane Niches and Digestive Tract of the Giant Borer

#### Bacteria From the Apoplast Fluid of Sugarcane Plants

The harvest of the apoplast fluid from the sugarcane stems for bacilli bacterial isolation followed the protocol described by Dong et al. (1994). Briefly, the stems were washed with neutral detergent and tap water after which the external layer was peeled off. The internodes were cut into pieces of approximately 5 cm and sterilized superficially by immersion in ethanol and rapid flaming. The internodes were then placed in Falcon tubes containing a 0.5 mL microtube, in order to avoid contact between the extracted fluid and the internode. The tubes were centrifuged at 3,000 *g* for 20 min at 20°C, conditions designed to avoid the extraction of liquid from the symplast. All fluid extract was removed from the tube with the aid of a pipette and then pasteurized.

#### Bacteria From the Central Cylinder Region of Sugarcane Internodes

The internodes used to isolate bacilli bacteria from the central cylinder region were the same as those used for the apoplast fluid extraction. Immediately after the extraction of the apoplast fluid, the outer part of the internodes was cut with a knife and discarded. The remaining central cylinder region was macerated with the aid of a mixer (IKA^®^, Model: A11 BS1) in saline solution (Baldani et al., 2014). The macerated materials were then pasteurized.

#### Bacteria From Sugarcane Roots

The sugarcane roots were initially washed under tap water and the excess water was removed with paper towels. Then, superficial root disinfestation was carried out according the protocol described by Baldani et al. (2014). All procedures were carried out in a cabinet flow in order to avoid contamination and the last water used for washing was plated in a culture medium to check if the sterilization had been satisfactory. After disinfestation, the roots were macerated with a mixer (IKA^®^, Model: A11 BS1) in saline solution (Baldani et al., 2014) and then pasteurized.

#### Bacteria From Digestive Tract of Giant Borer Larvae

**FIGURE 1:**
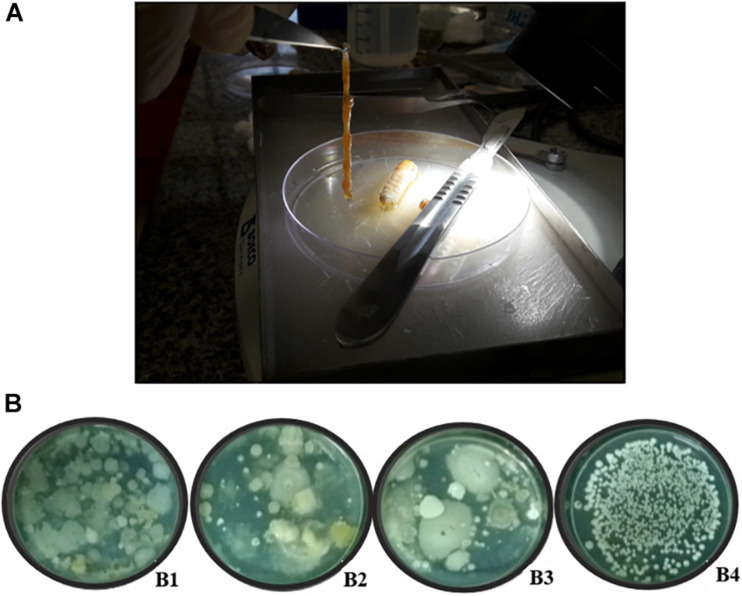
Digestive tract of giant borer (*Telchin licus licus*) and the diversity of colonies in solid BHI medium used for the isolation of bacteria. **(A)** The digestive tract being removed with the aid of forceps; **(B)** (B1) Bacteria obtained from the central region of the internodes, (B2) apoplast fluid, (B3) sugarcane roots, and (B4) digestive tract of the giant borer.

The larvae of the giant borer were killed by placing them in flasks containing wool embedded in chloroform. The larvae were then superficially disinfected following the protocol adapted from Orozco-Flores et al. (2017): immersion of larvae in sodium hypochlorite (2%) for 1 min followed by three successive washes with sterilized distilled water. To remove the digestive tract, the head and the last uromere of each larva were removed with a scalpel and the digestive tract was removed with the aid of a forceps, pulling it out from the larva (Figure 1A). The digestive tract was macerated with sterile saline solution (NaCl 0.85%) using a glass stick, followed by pasteurization.

#### Pasteurization Process and Isolation of Endospore-Forming Bacteria

To reduce the microbial Gram-negative population of the samples and to isolate mainly endospore-forming bacteria, aliquots of 1.0 mL from the apoplast fluid, the macerate suspension of the central internode region, and the roots were pasteurized following the World Health Organization protocol (World Health Organization [WHO], 1999). The aliquots were placed in 1.5 mL microtubes and submitted to a temperature of 80°C for 12 min followed by incubation on ice for 5 min.

The pasteurized samples were submitted to serial dilution (10° to 10^–2^) and aliquot of 100 μL each dilution was spread onto Petri dishes containing solid BHI medium with pH 7.0 (Brain Heart Infusion by Becton, Dickinson and Company^®^, United States). The plates were incubated at 30°C for 16 h. Bacteria with morphological characteristics like the *Bacillus* genus were streaked out onto cap tubes containing solid BHI slant medium, pH 7.0, incubated for 16 h at 30°C and stored under mineral oil for further characterization. To guarantee the isolation of only endospore-forming bacteria, the pasteurization process was carried out twice. However, in the second step of pasteurization, the bacteria isolated in the first step were grown in liquid BHI medium pH 7.0 at 30°C, 200 rpm for 16 h. After growth the bacterial culture was pasteurized and plated in solid BHI medium pH 7.0 and incubated at 30°C for 16 h. The isolates which had shown growth were again stored following the same procedure above. The Gram stain test (Gram, 1884) was performed with the isolates grown after the second pasteurization to confirm the Gram-positive characteristic of the endospore-forming bacteria.

### Molecular Analysis

#### DNA Extraction

DNA extraction from bacterial isolates was performed using the commercial kit Wizard^®^ Genomic DNA Purification (Promega Corporation, an affiliate of Promega Biotecnologia do Brasil, Ltda.) for chromosomal DNA extraction. The extracted DNA was quantified by fluorometry using the Qubit^TM^ dsDNA BR Assay kit (Q32853 - Thermo Fisher Scientific) and used as a template for the PCR amplification reactions (Polymerase Chain Reactions).

#### BOX-PCR Fingerprint of the Spore-Forming Bacteria

The BOX-PCR technique (genomic fingerprinting) applied to the isolates was carried out using A1R primer (Versalovic et al., 1994) which amplifies repetitive regions of the genome, and which is described in Table 1. The conditions used for amplifying the DNA of bacterial isolates were: 10 ng/μL of DNA, 1 mM of the primer, 2 X buffer, 3 mM MgCl_2_, 0.3 mM of dNTP and 5 U of GoTaq^®^ DNA polymerase (Promega), for a final volume of 25 μL. Incubation in the thermal cycler (SureCycler 8800, G8800A, Agilent Technologies, Inc., Santa Clara, CA, United States) was carried out under the following conditions: initial denaturation temperature of 95°C for 10 min, followed by 40 cycles of denaturation at 94°C for 1 min, annealing at 50°C for 1 min, and polymerization at 65°C for 8 min. At the end of the 40 cycles, the amplified product was submitted to a final extension temperature at 65°C for 16 min. The amplified DNA (10 μL) of the bacterial isolates was evaluated on agarose gel electrophoresis (2%) at 70 v, at 5 h. The amplified DNA band profiles were clustered using the BioNumerics software (Applied Maths NV.) and the dendrogram was generated using the unweighted pair group with the arithmetic mean (UPGMA) clustering method based on Jaccard coefficient (BioNumerics, Applied Maths NV.). The tolerance adopted to calculate the similarity coefficient of the bands was 3 and 1% optimization, since they were the parameters that best fit the isolate clustering.

**TABLE 1:** Primers used in polymerase chain reactions (PCR) to characterize and identify *Bacillus* species.

Analysis	Gene	Primers	Sequence	Amplified fragment size	References
Diversity (BOX-PCR)	Repetitive regions of genome (BOX)	A1R	5′-CTA CGG CAA GGC GAC GCT GAC G-3′	Various size	Versalovic et al., 1994
Biomarker amplification	XRE transcriptional regulator biomarker	XRE-F	5′-AAGATATTGCAAGCGGTAAGAT-3′	246	Wei et al., 2019
		XRE-R	5′-GTTTTGTTTCAGCATTCCAGTAA-3′		
Phylogenetic characterization	16S rRNA	27F	5′-AGA GTT TGA TCC TGG CTC AG-3′	1500	Furushita et al., 2003
		Amp2	5′-AAG GAG GTG ATC CAR CCG CA-3′		Wang et al., 1996
Housekeeping genes	gmk (guanylate kinase, putative)	gmkF	5′-ATTTAAGTGAGGAAGGGTAGG-3′	600	Jolley et al., 2018 (*Bacillus cereus* Multi Locus Sequence Typing website (https://pubmlst.org/bcereus/)
		gmkR	5′-GCAATGTTCACCAACCACAA-3′		
	*glpF* (glycerol uptake facilitator protein)	glpF-F	5′-GCGTTTGTGCTGGTGTAAGT-3′	549	
		glpF-R	5′-CTGCAATCGGAAGGAAGAAG-3′		
	*pycA* (pyruvate carboxylase)	pycA-F	5′-GCGTTAGGTGGAAACGAAAG-3′	550	
		pycA-R	5′-CGCGTCCAAGTTTATGGAAT-3′		
	*pta* (phosphate acetyltransferase)	pta-F	5′-GCAGAGCGTTTAGCAAAAGAA-3′	576	
		pta-R	5′-TGCAATGCGAGTTGCTTCTA-3′		
	*ilvD* (dihydroxy-acid dehydratase)	ilvD-F	5′-CGGGGCAAACATTAAGAGAA-3′	556	
		ilvD-R	5′-GGTTCTGGTCGTTTCCATTC-3′		
	*tpi* (triosephosphate isomerase)	tpi-F	5′-GCCCAGTAGCACTTAGCGAC-3′	558	
		tpi-R	5′-CCGAAACCGTCAAGAATGAT-3′		

#### Amplification and Sequencing of 16S rRNA Region

The encoding region of the 16S rRNA gene of Gram-positive bacterial isolates was amplified by PCR using two primer pairs, 27F (Furushita et al., 2003) and Amp2 (Wang et al., 1996) which are described in Table 1. The reagents were adjusted to 50 ng chromosomal DNA, 1 X buffer, 2.0 mM MgCl_2_, 0.2 mM dNTPs, 1 U GoTaq^®^ DNA polymerase (Promega) and 0.2 pMol of each primer in a final volume reaction of 50 μL. These reactions were incubated in the thermocycler (SureCycler 8800, G8800A, Agilent Technologies, Inc., Santa Clara, CA, United States) and the amplification conditions were: an initial denaturation temperature of 94°C for 3 min, followed by 29 cycles of denaturation at 94°C for 1 min, annealing at 65°C for 1 min and polymerization at 72°C for 5 min. At the end, the amplification was submitted to a final extension temperature of 72°C for 5 min. The amplified fragment was evaluated on agarose gel electrophoresis (0.7%) at 100 v for 1 h and the PCR product was sequenced in the 3500 Genetic Analyzer (Applied Biosystem^®^) automatic sequencer at the Embrapa Agrobiologia. The alignment of the sequences was performed using BioEdit software followed by comparing the sequences with those deposited in the database (Genbank database^1^), using the BLAST-N program (Altschul et al., 1997). The sequences were analyzed and a phylogenetic tree was built with the MEGA program v.7.0 program (Kumar et al., 2016), using the Neighbor-Joining (NJ) and Bootstrap statistical method of 1000 replicates, taking into account the differences and homologies between sequences in order to know the evolutionary history of the microorganisms.

#### Amplification of the Transcriptional Regulator XRE Biomarker

The XRE transcriptional regulator was recently tested to differentiate *B. thuringiensis* from other species within the *B. cereus* group (Wei et al., 2019). The authors succeeded in identifying *B. thuringiensis* species, with an accuracy of 97.5%. Therefore, the bacterial isolates of the *B. cereus* group (separated according to the 16S rRNA phylogenetic tree) were amplified using the primers designed by Wei et al. (2019) that are described in Table 1. The expected size of the amplified fragment was 246 bp. The PCR conditions for amplification of the XRE biomarker were: DNA concentration of 50 ng, 10 pmol of each primer, 1 U of GoTaq^®^ DNA polymerase, 250 μM of dNTP, 1 X buffer and 2 mM of MgCl_2_ for a final volume of 20 μL reaction. The amplification conditions were an initial denaturation temperature of 95°C for 5 min, followed by 35 cycles of denaturation at 95°C for 30 s, annealing at 47°C for 30 s and polymerization at 72°C for 30 s. At the end of the process, the amplification was submitted to a final extension temperature of 72°C for 5 min. The amplified fragment was run on agarose gel electrophoresis (1.5%) at 100 v for 2 h.

#### Amplification of Housekeeping *gmk*, *glp*, *pycA*, *pta*, *ilvD*, and *tpi* Genes

For a more refined separation of *B. thuringiensis* species from other species of the *B. cereus* group, analysis was performed using fragments of housekeeping genes (Multi Locus Sequence Typing – MLST). The selected housekeeping genes have already been published in the PubMLST bcereus database^2^ (Jolley et al., 2018) and the primers and the expected fragment size are described in Table 1.

The PCR condition was the same for all genes, using a DNA concentration of 20 ng, 10 pmol of each primer, 1 U of GoTaq^®^ DNA polymerase, 400 μM of dNTP, 1 X buffer and 2 mM of MgCl_2_ for a final reaction volume of 25 μL. The amplification conditions were an initial denaturation temperature of 95°C for 5 min, followed by 35 cycles of denaturation at 95°C for 30 s, specific annealing temperature for each primer pairs by 30 s (*gmk* at 56°C, *glpF* at 59°C, *pycA* at 57°C, *pta* at 56°C, *ilvD* and *tpi* at 58°C) and polymerization at 72°C for a minute. At the end, the amplification was submitted to a final extension temperature of 72°C for 10 min. The amplified fragment was run on agarose gel electrophoresis (1.5%) at 100 v for 2 h. The PCR product was sequenced in the 3500 Genetic Analyzer (Applied Biosystem^®^) automatic sequencer from the Embrapa Agrobiologia. The sequences were aligned, and the phylogenetic trees were built in the MEGA v.7.0 program (Kumar et al., 2016) using the Neighbor-Joining (NJ) statistical method and the evolutionary distances were calculated using the Tamura 3-parameter model. Sequences of species within the *Bacillus* genus deposited in the NCBI database were used to cluster the bacterial isolates within the dendrogram.

## Results

### Morphological Characterization

Great diversity of bacterial colony morphology was observed on BHI plate medium spread with the suspension of sugarcane plant tissues after exposure to the first pasteurization process (Figure 1B). In contrast, colonies from the giant borer digestive tract did not show visual morphological diversity (Figure 1B).

The second round of pasteurization carried out with the 796 purified strains which had been isolated after the first process, showed colonies with morphological characteristics of *Bacillus*. A total of 425 isolates were then selected, with 108 coming from the apoplast fluid, 175 from the central cylinder region of the internodes, 94 from surface disinfested roots, and 48 from the digestive tract of the giant borer. These 425 isolates were then subjected to Gram staining test and 161 isolates turned blue, which is a characteristic of Gram-positive bacteria, when submitted to phase contrast microscopic analysis. Of those, 46 were from the apoplast fluid, 46 from the central cylinder region of the internodes, 50 from surface disinfested roots and 19 from the digestive tract of the giant borer.

### Molecular Characterization

#### BOX-PCR Genomic Fingerprinting

The BOX-PCR fingerprinting analysis was performed for 157 Gram-positive isolates. Four isolates were not analyzed because they did not show product of amplification. The analysis showed the presence of 127 distinct band patterns. The number of detected bands varied from 1 to 28 and the size ranged from 100 to 5000 bp.

**FIGURE 2:**
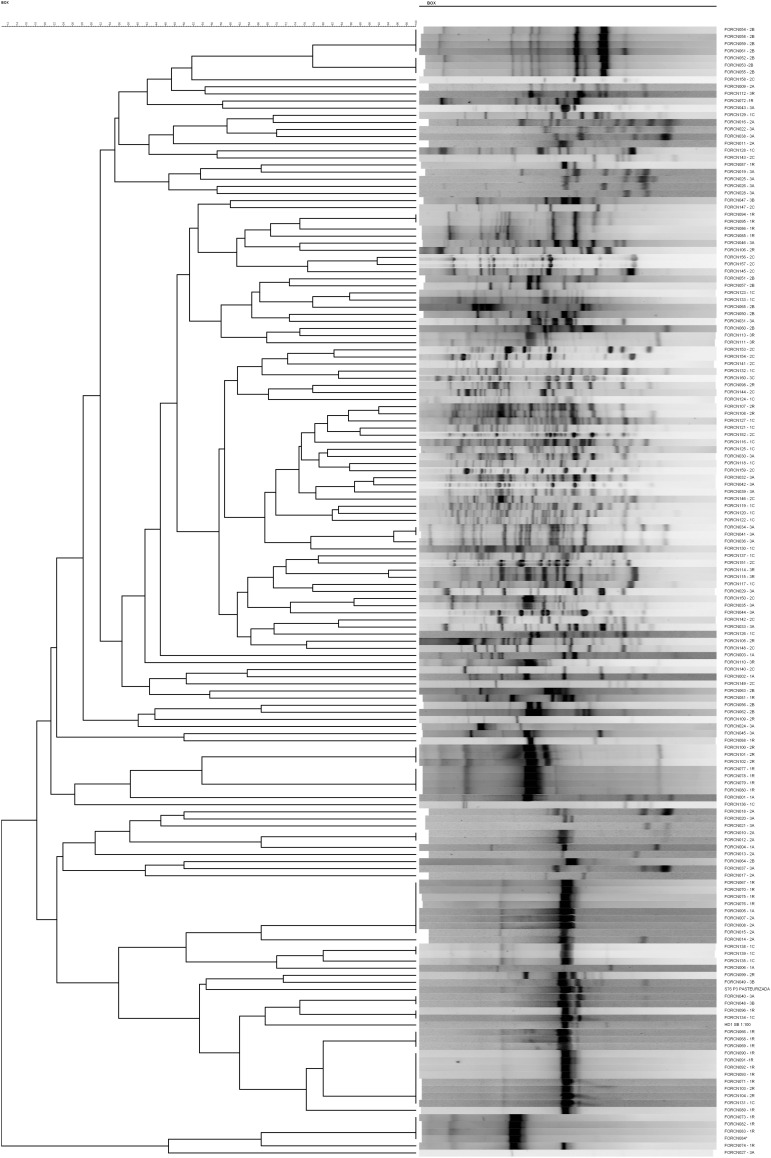
UPGMA similarity dendrogram of BOX-PCR fingerprinting. The dendrogram was generated of fingerprinting patterns of 157 bacterial isolates from sugarcane niches (apoplast, central region of the internodes, roots) and the digestive tract of the giant borer (*Telchin licus licus*).

Regarding the isolating niches, most bacteria did not show pattern profiles similar to those of other analyzed isolates, as demonstrated in the dendrogram (Figure 2). Therefore, these different band profiles indicate that the bacilli isolated from the sugarcane niches and giant borer digestive tract belong to different *Bacillus* species and/or subspecies.

#### 16S rRNA-Gene Base Analysis

The similarity analysis based on the 16S rRNA sequences was performed for 157 bacterial strains. The sequences were compared with sequences deposited in the GenBank enabled the isolates to be phylogenetically clustered into 22 species within the genus *Bacillus* (138 isolates) (Supplementary Table 2). Among the species identified, there were strains closely related to *B. aerius*, *B. albus*, *B. amyloliquefaciens*, *B. australimaris*, *B. cereus*, *B. circulans*, *B. drentensis*, *B. flexus*, *B. kochii*, *B. licheniformis*, *B. megaterium*, *B. nealsonii*, *B. oleronius*, *B. pacificus*, *B. proteolyticus*, *B. safensis*, *B. subterraneus*, *B subtilis*, *B. tequilensis*, *B. thuringiensis*, *B. velezensis* and *B. wiedmannii*. In addition, some isolates were phylogenetically clustered with other Gram-positive genera/species: *Brevibacillus invocatus* (one isolate), *Fictibacillus barbaricus* (one isolate), *Lysinibacillus cresolivorans* (one isolate), *L. fusiformis* (one isolate), *L. macroides* (four isolates), *Paenibacillus alvei* (one isolate), *P. barcinonensis* (one isolate), *P. illinoisensis* (six isolates), *P. vulneris* (one isolate), and *Terribacillus saccharophilus* (two isolates).

**FIGURE 3:**
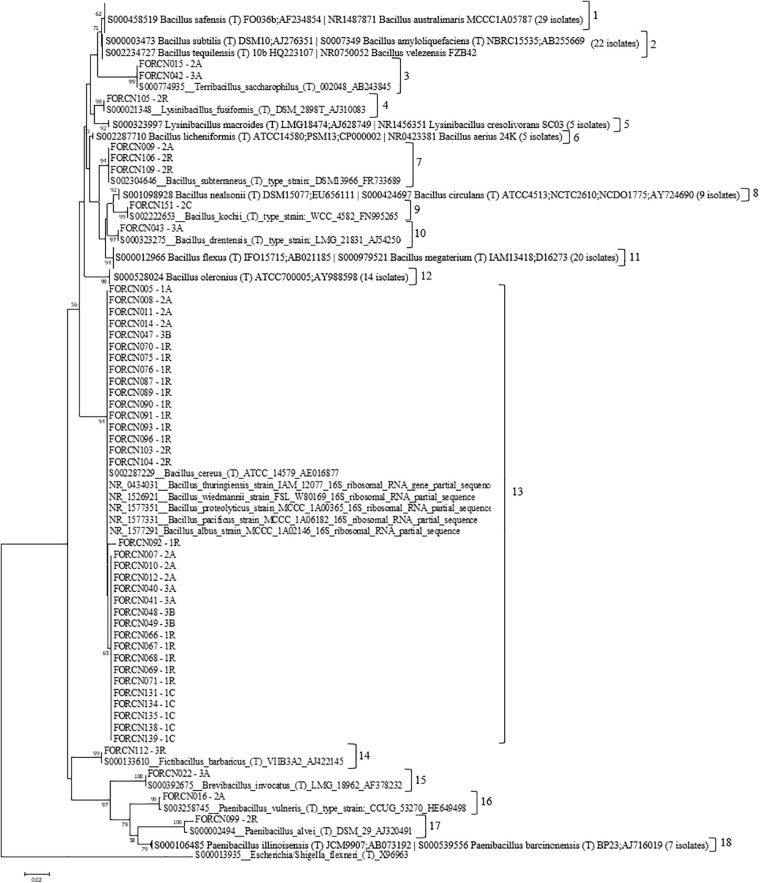
Phylogenetic relationships of bacteria isolated from different sugarcane niches estimated based on the 16S rRNA sequence. The tree was obtained using the Neighbor-Joining (NJ) statistical method and the Tamura 3-parameter model as indicated by the model test function of the MEGA 7.0 program. Bootstrap values are shown when the represented relationships have been observed in at least 50% of 1000 replicates. *Escherichia*/*Shigella flexneri* was used as an external group. The numbers 1–18 indicate the groups formed.

The built phylogenetic tree showed the bacterial strains clustered within 18 groups (Figure 3). Group 13 appeared to be the most interesting one since it was formed by 34 isolates phylogenetically related to species *B. cereus*, *B wiedmannii*, *B. proteolyticus*, *B. pacificus*, and *B. albus*. It also includes strains belonging to *B. thuringiensis* species, which has interesting characteristics related to the biological control of pests.

The most representative species were phylogenetically closely related to *B. megaterium* (11.5%) followed by *B. safensis* (10.8%), *B. cereus* (8.9%), *B. oleronius* (8.9%), *B. amyloliquefaciens* (7.0%), and *B. pacificus* (6.4%) (Table 2). Concerning the niche of bacterial isolation, it was observed that approximately 17.4% of strains, originated from the apoplast fluid, showed 99% similarity to *B. megaterium* sequences, 31.8% from the central cylinder region of the internodes showed 99% similarity to *B. oleronius* sequences, 22.9% from disinfested roots showed greater similarity (99%) to *B. amyloliquefaciens* sequences and 47.4% obtained from the giant borer digestive tract showed 99/100% similarity to *B. safensis* sequences.

**TABLE 2:** Number of isolates representing species and percentage of species in all niches.

Identified species	Number of species per niche				Number of species in all niches	Species percent (%)
	Apoplast fluid	Roots	Central cylinder region of the internodes	Digestive tract of the giant borer		
*Bacillus aerius*	7	0	0	0	7	4.5
*Bacillus albus*	2	0	4	0	6	3.8
Bacillus amyloliquefaciens*	0	11	0	0	11	7.0
*Bacillus australimaris*	1	0	0	5	6	3.8
Bacillus cereus*	4	6	1	3	14	8.9
*Bacillus circulans*	0	0	8	0	8	5.1
*Bacillus drentensis*	1	0	0	0	1	0.6
*Bacillus flexus*	0	2	0	0	2	1.3
*Bacillus kochii*	0	0	1	0	1	0.6
*Bacillus licheniformis*	4	0	1	0	5	3.2
Bacillus megaterium*	8	7	3	0	18	11.5
*Bacillus nealsonii*	0	0	1	0	1	0.6
Bacillus oleronius*	0	0	14	0	14	8.9
Bacillus pacificus*	0	10	0	0	10	6.4
*Bacillus proteolyticus*	1	1	0	0	2	1.3
Bacillus safensis*	5	1	2	9	17	10.8
*Bacillus subterraneus*	1	2	0	0	3	1.9
*Bacillus subtilis*	1	2	0	0	3	1.9
*Bacillus tequilensis*	0	3	0	0	3	1.9
*Bacillus thuringiensis*	1	0	0	0	1	0.6
*Bacillus velezensis*	0	0	4	0	4	2.5
*Bacillus wiedmannii*	1	0	0	0	1	0.6
*Brevibacillus invocatus*	1	0	0	0	1	0.6
*Fictibacillus barbaricus*	0	1	0	0	1	0.6
*Lysinibacillus cresolivorans*	0	0	1	0	1	0.6
*Lysinibacillus fusiformis*	0	1	0	0	1	0.6
*Lysinibacillus macroides*	0	0	4	0	4	2.5
*Paenibacillus alvei*	0	1	0	0	1	0.6
*Paenibacillus barcinonensis*	0	0	0	1	1	0.6
*Paenibacillus illinoisensis*	5	0	0	1	6	3.8
*Paenibacillus vulneris*	1	0	0	0	1	0.6
*Terribacillus saccharophilus*	2	0	0	0	2	1.3
Total/niche	46	48	44	19	157	

*Most representative species.

**FIGURE 4:**
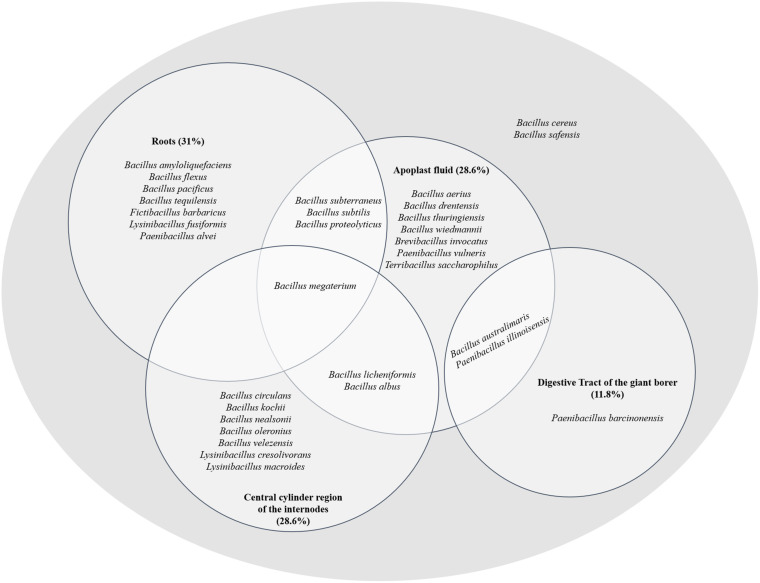
Species phylogenetically identified in the different sugarcane niches (apoplast, central cylinder region of the internodes and roots) and in the digestive tract of the giant borer (*Telchin licus licus*). The diagram shows species inhabiting different niches.

The Venn diagram demonstrated that some bacterial genera were exclusive to certain niches, such as *Brevibacillus* and *Terribacillus*, isolated from the apoplast fluid and the genus *Fictibacillus* originated from roots (Figure 4). On the other hand, some bacterial species are capable of inhabiting different niches, such as *B. subterraneus*, *B. subtilis*, *B. proteolyticus*, *B. megaterium*, *B. licheniformis*, and *B. albus* isolated either from two or three sugarcane niches (apoplast fluid, central cylinder of the internode region and roots) (Figure 4).

Two species, *B. australimaris* and *Paenibacillus illinoisensis*, were able to inhabit the apoplast and digestive tract of the giant borer (Figure 4), showing the ability to adapt to quite different conditions. In addition, the species *B. cereus* and *B. safensis* were found in all niches (Figure 4).

**FIGURE 5:**
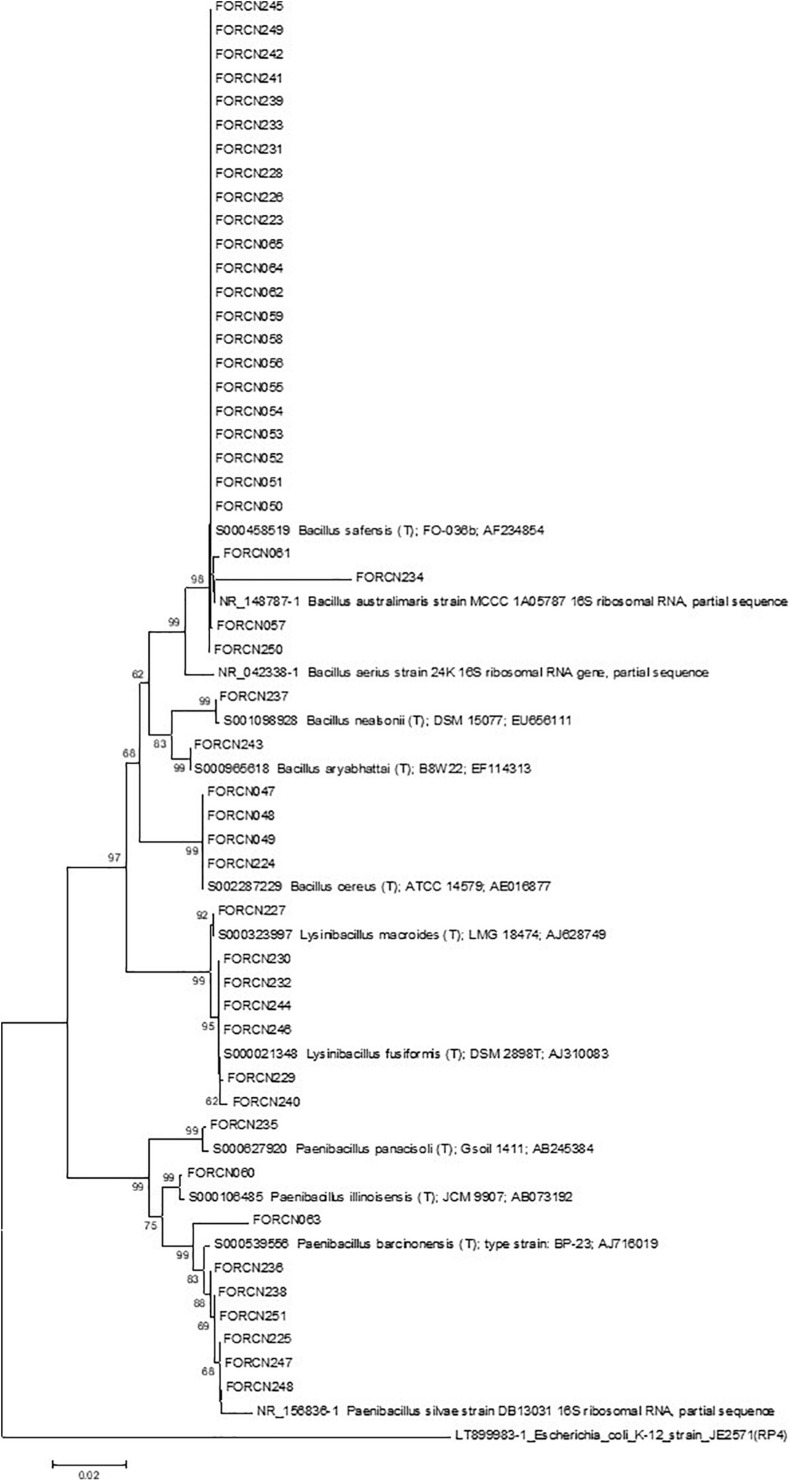
Phylogenetic relationships of isolated bacteria from the digestive tract of the giant borer estimated by 16S rRNA sequence. The tree was obtained using the Neighbor-Joining (NJ) statistical method and the Kimura 2-parameter model as indicated by the model test function of the MEGA 7.0 program. Bootstrap values are shown when the represented relationships have been observed in at least 50% of 1000 replicates. The scale bar represents the number of base pair substitutions per site. *Escherichia coli* was used as an external group.

The phylogenetic characterization of the bacterial strains showed that all sugarcane niches presented high diversity at the species level, correlated with many species of bacteria. The apoplast fluid correlated with 17 species which are phylogenetically grouped in the genera *Bacillus*, *Brevibacillus*, *Paenibacillus*, and *Terribacillus*. The roots and the central region have bacteria which are phylogenetically related to 13 and 12 species respectively (Supplementary Table 2). In contrast to the sugarcane niches, the digestive tract of the giant borer showed only five Gram-positive bacterial species colonizing the larvae. These strains were phylogenetically related to species *B. australimaris*, *B. cereus*, *B. safensis*, *P. barcinonensis, and P. illinoisensis* (Supplementary Table 2). In addition, the bacteria from the giant borer digestive tract that presented Gram-negative characteristics after the second pasteurization process, were also included in the taxonomical characterization. It was observed that all these bacteria showed high phylogenetic similarity to 12 different bacilli species: *B. aerius*, *B. aryabhattai*, *B. australimaris*, *B. cereus*, *B. nealsonii*, *B. safensis*, *P. barcinonensis*, *P. illinoisensis*, *P. panacisoli*, *P. silvae*, *L. fusiformis*, and *L. macroides* (Supplementary Table 3). Therefore, these results suggest that all strains are Gram-positive, despite the results of the Gram-negative cell wall test. A phylogenetic tree with these bacterial strains from the digestive tract of the giant borer was constructed and eight groups were then formed (Figure 5). The same species with Gram-positive and Gram-negative stains were grouped together (Figure 5), which suggests that the same species can present variable Gram staining results.

#### XRE *B. thuringiensis* PCR Biomarker

**FIGURE 6:**
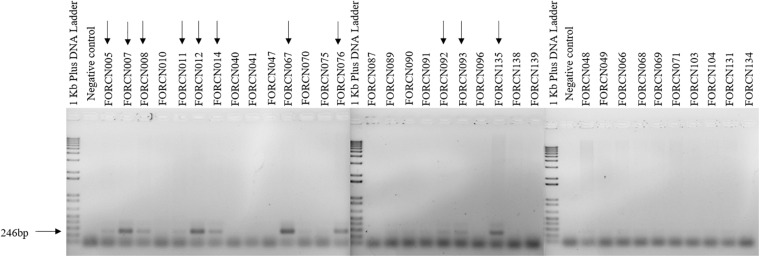
Agarose gels (1.5%) showing the 246 bp PCR amplification product amplified using the specific primers for the XRE transcriptional regulator. The arrows indicate the strains with a positive result.

The PCR amplification of the XRE transcriptional regulator with *B. thuringiensis* specific primers revealed that, among the 34 bacterial strains phylogenetically related to *B. cereus* group, 11 strains (FORCN005, 007, 008, 011, 012, 014, 067, 076, 092, 093, and 135) presented amplified product of the expected size (∼246 bp) as shown in Figure 6. Based on these results, the strains FORCN005, 007, 008, 011, 012, 014, 067, 076, 092, 093, and 135 were tentatively included in the *B. thuringiensis* species. All these strains have the capacity to induce the spore formation during growth for 72 h in HCT medium (data not shown). The other strains (FORCN010, 040, 041, 047, 048, 049, 066, 068, 069, 070, 071, 075, 087, 089, 090, 091, 096, 103, 104, 131, 134, 138, and 139) presented neither one band nor bands of different sizes and therefore may belong to different species.

A comparison of the XRE transcriptional regulator results with the dendrogram generated by the BOX-PCR pattern profiles showed that strains FORCN005, 007, 008, 012, 014, 067, 076, 092, 093, and 135 presented very similar band patterns (Figure 2), forming a group of bacilli or part of a closely related group.

#### Housekeeping *Bacillus* Gene Analysis

Amplification of conserved housekeeping genes allowed different trees to be built and therefore the phylogenetic evolution of strains within the *B. cereus* group could be visualized. For example, analysis of the phylogenetic trees generated with the *glp* (Supplementary Figure 1), *gmk* (Supplementary Figure 2), and *pta* (Supplementary Figure 3) gene sequences indicated that strains FORCN075, 076, 089, 090, 091, 092, and 093 clustered together with the species *B. thuringiensis* serovar *berliner*. The FORCN070 and 096 strains were also clustered within the *B. thuringiensis* serovar *berliner* strain, but the study was based only on the *glp* and *gmk* gene trees (Supplementary Figures 1, 2).

The tree generated with *tpi* gene sequences showed that many strains: FORCN005, 007, 008, 010, 011, 012, 014, 047, 048, 049, 068, 070, 071, 075, 076, 089, 090, 091, 092, 093, 096, 103, 104, and 131 were clustered within *B. thuringiensis* subsp. *kurstaki* S76, *B. thuringiensis* subsp. *kurstaki* HD-1 and *B. cereus* (Supplementary Figure 4). Tree analysis with *pycA* gene also grouped the strains FORCN010, 011, 012, 048, and 049 with *B. thuringiensis* subsp. *kurstaki* strains S76 and HD-1 (Supplementary Figure 5). Furthermore, the phylogenetic tree constructed with sequences of the conserved *ilvD* gene, showed that the strains FORCN010, 049 and 104 were clustered with the species of *B. thuringiensis* subsp. *kurstaki* S76 (Supplementary Figure 6).

**FIGURE 7:**
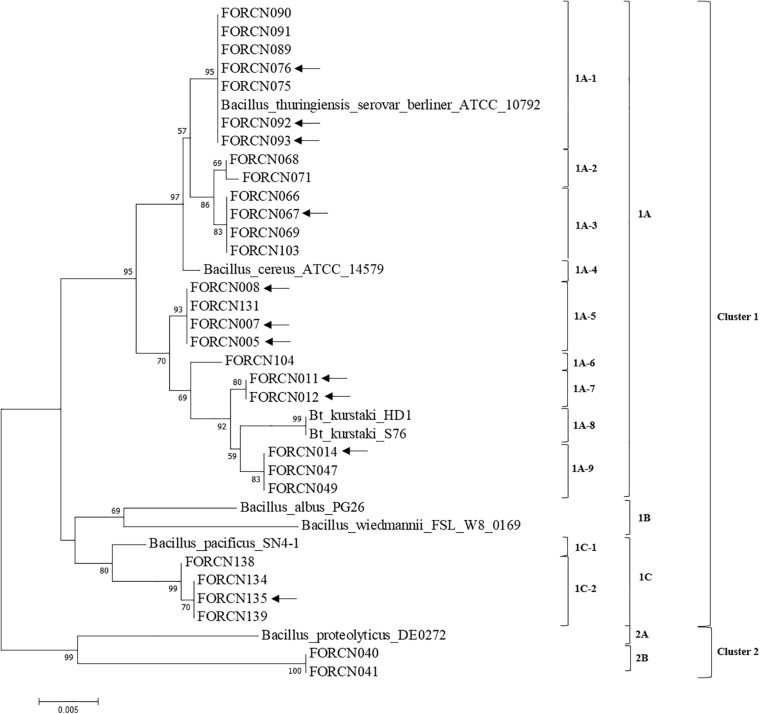
Phylogenetic tree using the Neighbor-Joining method estimated from the concatenated sequences of four conserved genes (*glp*, *gmk*, *pta*, and *tpi*). The evolutionary distances were calculated using the Tamura 3-parameter model as indicated by the model test function of the MEGA 7.0 program. Bootstrap values are shown when the relationships represented have been observed in at least 50% of 500 replicates. The scale bar represents the number of base pair substitutions per site. Sequences of species of the genus *Bacillus* deposited in the NCBI database were used to group the bacterial isolates in the dendrogram. The arrows indicate the strains with positive results for the amplification of XRE transcriptional regulator.

The concatenated phylogenetic tree analysis based on the 943 nucleotides of four conserved genes (*glp*, *gmk*, *pta*, and *tpi*), showed two phylogenetic clusters (Cluster 1 and Cluster 2). Cluster 1 contained 27 isolates and cluster 2 was formed by just two isolates (FORCN040 and 041) clustering with *B. proteolyticus* strain (Figure 7). Cluster 1 was divided into three subgroups (subgroup 1A, 1B, and 1C) and within these subgroups, smaller subgroups were identified (Figure 7). In subgroup 1A, the isolates (FORCN005, 007, 008, 011, 012, 014, 047, 049, 066, 067, 068, 069, 071, 075, 076, 089, 090, 091, 092, 093, 103, 104, and 131) were grouped with strains of *B. cereus*, *B. thuringiensis* serovar *berliner* and *B. thuringiensis* subsp. *kurstaki*. The strains *B. wiedmannii* and *B. albus*, used as type strains, were grouped in subgroup 1B. Subgroup 1C, on the other hand, included isolates FORCN134, 135, 138, 139 and strain *B. pacificus*. A few strains were not included in these analyses because the sequences were neither of high quality nor amplified for certain housekeeping genes.

The strains FORCN005, 007, 008, 011, 012, 014, 067, 076, 092, 093, and 135 that presented positive PCR product for the transcriptional regulator XRE (corresponding to the *B. thuringiensis* species) may be considered different *Bacillus* subspecies or serovars. According to the conserved gene analysis, these strains were clustered in the same group (Cluster 1), but in different subgroups in the evolutionary trees for the different genes (Figure 7).

## Discussion

Endophytic microorganisms have numerous functional characteristics that make them interesting targets for agricultural applications. In this work, a great number of endophytic bacilli bacteria were isolated from sugarcane tissues and the digestive tract of the giant borer, a pest that causes severe damage to sugarcane plants (Linares et al., 1996). Even though these isolated bacteria were subjected to the pasteurization process twice (World Health Organization [WHO], 1999), many cells still had a reddish color, a characteristic color of Gram-negative bacteria in the Gram staining test (Gram, 1884). It has been observed that the Gram staining test varies with bacterial culture age, that is, at the beginning of growth they are Gram-positive but older cultures are variable (Mo et al., 2020). Other authors have also identified *Bacillus* species with variable stain (Keita et al., 2013; Rocha et al., 2017), indicating that it is not possible to use only this test for characterization of bacilli microorganisms, since it can generate false negative results.

There are several works in the literature that confirm the presence of *Bacillus* species living inside plants, such as the species *B. toyonensis*, *B. megaterium*, *B. cereus*, *B. aryabhattai*, *B. stratosphericus*, and *B. aerius* that were identified in association with tomato plants in Brazil (Rocha et al., 2017). Karthik et al. (2017) also observed the presence of *Bacillus* species in association with banana cultivars from three regions of India (*B. tequilensis*, *B. subtilis*, *B. methylotrophicus*, *B. megaterium*, and *B. aryabhattai*). In a work carried out by Kushwaha et al. (2020), *B. amyloliquefaciens*, *B. subtilis* and *B. cereus* were identified in association with *Pennisetum glaucum* plants grown in India. The results obtained by Karthik et al. (2017); Rocha et al. (2017), and Kushwaha et al. (2020) reinforce the importance of endophytic *Bacillus* strains for the biocontrol and plant growth promotion application because they have several functional characteristics such as synthesis of 3-acetic acid, siderophore, hydrolytic enzyme activities and the ability to solubilize inorganic phosphate and zinc.

The endophytic community of *Bacillus* associated with different plants is quite diverse and a similar result was obtained in our work, confirmed by the molecular analyses of Gram-positive bacilli isolates based on the 16S rRNA gene sequences. This analysis allowed the 157 bacterial strains to be clustered, at the species level, within 32 species isolated from the sugarcane niches (apoplast fluid, central internodes region and roots) and 12 species from the digestive tract of the giant borer.

The apoplast fluid was the niche with the highest number of bacterial species, 17 species identified among the genera *Bacillus*, *Brevibacillus*, *Paenibacillus*, and *Terribacillus*. The reason for this high species diversity is unknown, although the amount of nutrients available in the internal tissues of sugarcane may have favored the establishment of microorganisms (Karthik et al., 2017). Among the strains, the species *B. megaterium* was the most representative, according to the taxonomical characterization (16S rRNA region), followed by species closely related to *B. safensis*, *B. cereus*, *B. oleronius*, *B. amyloliquefaciens*, and *B. pacificus*.

A great diversity of endophytic *Bacillus* species in sugarcane has already been identified in a work developed by Melo et al. (2021), that isolated eight species of *Bacillus* (*B. cereus*, *B. megaterium*, *B. flexus*, *B. aryabhattai*, *B. safensis*, *B. altitudinis*, *B. velezensis*, and *B. thuringiensis*) and *Paenibacillus taichungensis* from apoplast fluid of sugarcane grown in Brazil. These authors also found that the species *B. megaterium* was the most representative and found that some strains promoted biomass accumulation in sugarcane plants and have antagonistic activity against sugarcane phytopathogenic fungi in *in vitro* assays. The endophytic species *B. amyloliquefaciens*, *B. aryabhattai*, *B. safensis*, *B. aerophilus*, and *B. subtilis* have been isolated from sugarcane roots grown in Thailand (Kruasuwan and Thamchaipenet, 2016) and the species *B. subtilis*, *B. cereus* and *B. pumilus* were isolated from the apoplast fluid of sugarcane grown in Cuba (Velázquez et al., 2008). Recently, eleven endophytic *Bacillus* species such as *B. luciferensis*, *B. aryabhattai*, *B. altitudinis*, *B. tequilensis*, *B. megaterium*, *B. acidiceler*, *B. pacificus*, *B. solisilvae*, *B. velezensis*, *B. tropicus*, and *B. australimaris* were found to be associated with sugarcane roots grown in China (Wang et al., 2019). All these studies point out the importance of the interaction of endophytic microorganisms with plants, since they promote plant growth and have interesting characteristics for biocontrol of pests and diseases.

The starting point for selection of *Bacillus* strains envisaging future biotechnological application to control sugarcane pests is the taxonomical identification of the bacterial species mainly considering the difficulties to separate species within the *Bacillus cereus* and *B. thuringiensis* clade even when complete genomic sequences are compared (Baek et al., 2019). Here, we use different molecular identification methods to separate *B. thuringiensis* species from closely related *Bacillus cereus* group (Okstad and Kolsto, 2012). For example, we amplified repetitive regions within the genome, a technique that has been used to compare isolates and understand the relationship between strains (Versalovic et al., 1994). The BOX-PCR fingerprinting from our Gram-positive isolates showed diversified profiles. Most of the bacterial isolates with similar phylogenetic characterization (16S rRNA) presented different band profiles in the fingerprinting analysis and were grouped differently in the dendrogram. The fingerprinting analysis for these strains indicated that they belong to other species or subspecies of *Bacillus*. Nevertheless, the results of the fingerprint analysis of some strains corroborated the phylogenetic characterization (16S rRNA) and were kept clustered together.

Comparison of the BOX-PCR fingerprinting of the *B. cereus* group (16S rRNA) showed that most of these strains presented a low number of polymorphic bands and were clustered in the same group or closely related groups in the dendrogram (Figure 2). The low number of polymorphic bands suggests that this may be a genomic characteristic of strains within the *B. cereus* group isolated in this study. Cherif et al. (2007) used ERIC and BOX-PCR techniques to separate strains of *B. thuringiensis* from *B. cereus* and found that some strains presented few amplified bands, such as the *B. thuringiensis* strain HD-1. In our work, the HD-1 strain also presented a low number of bands, which may be a characteristic of the bacterial strain.

A recent work showed that it is possible to distinguish the species *B. thuringiensis* from *B. cereus* using the XRE transcriptional regulator, which is able to control the production of most protein crystals produced by *B. thuringiensis* (Wei et al., 2019). According to the authors, it is possible to differentiate the two species with 97.5% accuracy. Comparing this methodology with that which uses the detection of *cry2* gene, the authors found that some *B. cereus* strains presented the c*ry2* gene, suggesting that the presence of the *cry* gene is not enough to separate the species from the *B. thuringiensis* which is known to carry *cry* genes (Wei et al., 2019). Our analyses using the XRE biomarker allowed 11 strains (FORCN005, 007, 008, 011, 012, 014, 067, 076, 092, 093, and 135) to be clustered within the *B. cereus* group, phylogenetically related to *B. thuringiensis* species. It is worth mentioning that the strain FORCN011 may represent a distinct subspecies since it is more distant from the other strains within the dendogram (Figure 2). Interesting, the taxonomic characterization based on the 16S rRNA region indicated that only the FORCN005 strain could be clustered to the species *B. thuringiensis* (Supplementary Table 2). This result reinforces the idea that the later methodology is insufficient to distinguish both species within the *B. cereus* group and that the transcriptional regulator XRE biomarker seems to be a good complementary technique for identification of the *B. thuringiensis* species.

The application of the conserved housekeeping genes in the phylogenetic analysis has been shown to be a powerful tool to separate *Bacillus* strains at the serovar level (Wang et al., 2018). A concatenated tree built based on the sequences of four conserved genes (*glp*, *gmk*, *pta*, and *tpi*) showed correspondence of the most analyzed isolates with subgroup 1A (which comprised the species *B. thuringiensis* serovar *berliner*, *B. thuringiensis* subsp. HD-1 and S76 and *B. cereus*). When the results are compared with those obtained with XRE biomarker and concatenated phylogenetic tree, the strains identified with *B. thuringiensis* according to XRE biomarker (FORCN005, 007, 008, 011, 012, 014, 067, 076, 092, 093, and 135) may be considered different *Bacillus* subspecies or serovars, since according to the result of the housekeeping genes they were clustered in different subgroups. In those cases where the taxonomic species identification are dubious, the solution would be to sequence the whole genome of the strains and use the Average Nucleotide Identity (ANI) as a tool to identify the *Bacillus* species (Jain et al., 2018).

The presence of microorganisms in the digestive tract of insect larvae was already known (Hammer et al., 2017), but the role played inside the lepidopteran larvae is still not well understood. The digestive tract of the giant borer has an alkaline pH of around 9.5, much higher than that found inside the sugarcane stem (juice with pH ∼5.5), a niche where the giant borer larvae move up and down and get the food. The alkaline pH of the digestive tract may hinder the establishment and survival of microorganisms (Bravo et al., 2013; Hammer et al., 2017), except for few groups of bacteria, including the *Bacillus* ones, that have a great ability to survive in an extreme environment in latency as endospores (Bobrowski et al., 2003). Recently, Paniagua Voirol et al. (2018) observed that the *Bacillus* genus was among the bacilli genera most frequently found in association with lepidopterans, and was present in more than 70% of 30 analyzed species of lepidopterans.

Similarly, many bacterial species (Gram-positive and negative) have been isolated from the digestive tract of *Spodoptera exigua* larvae (*Lysinibacillus macroides* and *L. fusiformis*; *Paenibacillus tylopili*, *P. amylolyticus* and *P. xylanexedens*; *Bacilus cereus*, *B. toyonensis* and *B. thuringiensis* strains Se13 and Se14) (Eski et al., 2018), a pest that causes losses in cotton, wheat, cabbage, and beet (Saeed et al., 2010). Our study showed the presence of twelve closely related species including *B. cereus*, *B. australimaris*, *B. safensis*, *B. nealsonii*, *B. aryabhattai*, *B. aerius*, *P. illinoisensis*, *P. barcinonensis*, *P. silvae*, *P. panacisoli*, *L. macroides*, and *L. fusiformis* colonizing the digestive tract of giant borer lepidopteran larvae. The entomopathogenic potential of these strains was not tested although it is already known that *B. thuringiensis* have the ability to control larvae of *D. saccharalis* (Gitahy et al., 2007), *S. frugiperda* (Monnerat et al., 2003), *P. xylostella* (Monnerat et al., 2009), *A. gossypii* (Melatti et al., 2010), and the species *Brevibacillus laterosporus* have been shown to be toxic to the diamondback moth (Ormskirk et al., 2019). Another species that has been observed with activity against *Culex quinquefasciatus* is *Lysinibacillus sphaericus* (Karungu et al., 2018). These studies suggest that different species or bacterial genera have the potential for biological control application. Therefore, further studies should focus not only in the *B. thuringiensis* species but also in other *Bacillus* species that may produce different entomopathogenic protein compounds that are toxic to giant borer.

In our work, all isolates belonged to the phylum Firmicutes. Bacteria from Firmicutes (59%) were also isolated from the gut of *S. littoralis* (Lepidoptera: Noctuidae) in early instar larvae and 97% in the advanced instar (Chen et al., 2016). Orozco-Flores et al. (2017) also identified the phyla Proteobacteria (74.4%), Firmicutes (9%), and Actinobacteria (14%) in the intestine of the lepidopteran *Plodia interpunctella*. It is believed that the pasteurization process applied here contributed to the isolation of only one phylum in contrast to what occurred in the other studies which used a different approach and isolated bacteria from other phyla in symbiosis with the host insects.

The presence of bacteria inside the larvae may be due to the ingestion of the diet which would maintain the habitats of these bacteria. Symbiotic bacteria found in the intestinal microbiota can be transmitted horizontally, through the host plant, and vertically, at the egg stage (Paniagua Voirol et al., 2018). In our case, the giant borer enters through the root base and moves up inside the stem by gnawing the cane stalk (Negrisoli Junior et al., 2015). Therefore, any bacteria colonizing the internal tissues should pass by the intestinal digestive tract of the larvae including the endophytic *Bacillus* species.

It is known that symbiotic microorganisms present in the intestines of insects promote several benefits for the host insect, such as improved nutrient absorption, protection against pathogens, protection against pesticides and the secondary metabolites produced by plants (Kikuchi et al., 2012; Engel and Moran, 2013; Salem et al., 2015; van den Bosch and Welte, 2016; Xia et al., 2017). Thus, the knowledge of the intestinal microbiota, as well as the functions played by these microorganisms can lead to more effective strategies for the biological control of insect pests, since the intestinal microbiota influences the biology of the insect (Paniagua Voirol et al., 2018). It has been observed that the absence of gut bacteria decreases the toxicity of *B. thuringiensis* against *Plodia interpunctella* and therefore the manipulation of gut bacteria could be a novel method of pest control (Orozco-Flores et al., 2017). So far, it is completely unknown the mechanism involved in the symbiosis of giant borer and the *Bacillus* intestinal microflora. Our future biocontrol strategy is to use a highly delta-endotoxin producing *Bacillus* strain capable to colonize endophytically the sugarcane plant tissues (roots and culms). The hypothesis is that the dying process begin when the larvae ingests the sugarcane tissues containing the entomophatogenic microorganism so that the pro-toxin is immediately solubilized due to the high pH and reducing conditions of insect gut, a mechanism already reported by Bravo et al. (2017).

## Conclusion

Our results showed that the distinction of closely related *Bacillus* species such as *B. thuringiensis* and *B. cereus* is extremely difficult, being indicated to use more than one methodology. The 16S rRNA sequencing region was important for identification at the genus level and provided hints about the possibly isolated species. Nevertheless, the most suitable methodology to separate species within the *B. cereus* group was the amplification of the XRE biomarker and sequencing of the housekeeping genes (*glp*, *gmk*, *pta*, and *tpi)*. These tools allowed us to consider the strains FORCN005, 007, 008, 011, 012, 014, 067, 076, 092, 093, and 135 as belonged to the *B. thuringiensis* species. However, it should be emphasized that the complete genomic sequence analysis of representative strains within each clade may provide a better view of the occurring *Bacillus* species within the sugarcane plant tissues and the living giant borer larvae.

The identification of these *Bacillus* species showed how complex is the symbiosis between plant-microorganism, with a diversified number of species living in association with sugarcane plants. We should keep in mind that this study did not consider non-cultivable bacterial species; therefore the number of species could be much higher than those identified here. By knowing that these microorganisms live inside the plants and in symbiosis with the target pest insects, it is essential to outline new strategic lines of research envisaging the development of bioproduct based on these *B. thuringiensis* strains to control sugarcane pests, mainly the giant borer.

## Data Availability Statement

The data presented in the study are deposited in the National Center for Biotechnology Information (NCBI) repository, accession numbers MW363203 to MW363359 and MW402865 to MW402893.

## Author Contributions

FR contributed to investigation, methodology, roles, and writing original draft preparation. AN and JB contributed to conceptualization, resources, and funding acquisition. JB also supervised this research. GM performed the validation of software analysis. PG and CR contributed with methodology. MV and JB curated data, writing, reviewing, and editing the manuscript. All the authors contributed to manuscript and approved the submitted version.

## Conflict of Interest

The authors declare that the research was conducted in the absence of any commercial or financial relationships that could be construed as a potential conflict of interest.

## Footnotes

1 http://www.ncbi.nlm.nih.gov

2 https://pubmlst.org/organisms/bacillus-cereus/primers
