# Supplementary material for: Endophytic Bacillus Bacteria Living in Sugarcane Plant Tissues and Telchin licus licus Larvae (Drury) (Lepidoptera: Castniidae): The Symbiosis That May Open New Paths in the Biological Control
Source: Front Microbiol. 2021 May 12;12:659965. doi: 10.3389/fmicb.2021.659965 (PMC8153187; doi:10.3389/fmicb.2021.659965)
Supplement: Supplementary file 1 [file Data_Sheet_1.docx]

***Supplementary Material***

**Supplementary Figures**

**Supplementary Figure 1** Phylogenetic tree using the Neighbor-Joining method estimated from 440 nucleotide positions of the *glp* gene. The evolutionary distances were calculated using the Tamura 3-parameter model as indicated by the model test function of the MEGA 7.0 program. Bootstrap values are shown when the relationships represented have been observed in at least 50% of 500 replicates. The scale bar represents the number of base pair substitutions per site. Sequences of species of the genus *Bacillus* deposited in the NCBI database were used to group the bacterial isolates in the dendrogram.

**Supplementary Figure 2** Phylogenetic tree built using the Neighbor-Joining method estimated from 443 nucleotide positions of the *gmk* gene. The evolutionary distances were calculated using the Tamura 3-parameter model as indicated by the model test function of the MEGA 7.0 program. Bootstrap values are shown when the relationships represented have been observed in at least 50% of 500 replicates. The scale bar represents the number of base pair substitutions per site. Sequences of species of the genus *Bacillus* deposited in the NCBI database were used to group the bacterial isolates in the dendrogram.

**Supplementary Figure 3** Phylogenetic tree using the Neighbor-Joining method estimated from 498 nucleotide positions of the *pta* gene. The evolutionary distances were calculated using the Tamura 3-parameter model as indicated by the model test function of the MEGA 7.0 program. Bootstrap values are shown when the relationships represented have been observed in at least 50% of 500 replicates. The scale bar represents the number of base pair substitutions per site. Sequences of species of the genus *Bacillus* deposited in the NCBI database were used to group the bacterial isolates in the dendrogram.

**Supplementary Figure 4** Phylogenetic tree using the Neighbor-Joining method estimated from 453 nucleotide positions of the *tpi* gene. The evolutionary distances were calculated using the Tamura 3-parameter model as indicated by the model test function of the MEGA 7.0 program. Bootstrap values are shown when the relationships represented have been observed in at least 50% of 500 replicates. The scale bar represents the number of base pair substitutions per site. Sequences of species of the genus *Bacillus* deposited in the NCBI database were used to group the bacterial isolates in the dendrogram.

**Supplementary Figure 5** Phylogenetic tree using the Neighbor-Joining method estimated from 480 nucleotide positions of the *pycA* gene. The evolutionary distances were calculated using the Tamura 3-parameter model as indicated by the model test function of the MEGA 7.0 program. Bootstrap values are shown when the relationships represented have been observed in at least 50% of 500 replicates. The scale bar represents the number of base pair substitutions per site. Sequences of species of the genus *Bacillus* deposited in the NCBI database were used to group the bacterial isolates in the dendrogram.

Supplementary Figure 6 Phylogenetic tree using the Neighbor-Joining method estimated from 541 nucleotide positions of the *ilvD* gene. The evolutionary distances were calculated using the Tamura 3-parameter model as indicated by the model test function of the MEGA 7.0 program. Bootstrap values are shown when the relationships represented have been observed in at least 50% of 500 replicates. The scale bar represents the number of base pair substitutions per site. Sequences of species of the genus *Bacillus* deposited in the NCBI database were used to group the bacterial isolates in the dendrogram.
