# Supplementary material for: Endophytic Bacillus Bacteria Living in Sugarcane Plant Tissues and Telchin licus licus Larvae (Drury) (Lepidoptera: Castniidae): The Symbiosis That May Open New Paths in the Biological Control
Source: Front Microbiol. 2021 May 12;12:659965. doi: 10.3389/fmicb.2021.659965 (PMC8153187; doi:10.3389/fmicb.2021.659965)
Supplement: Supplementary file 2 [file Data_Sheet_2.docx]

***Supplementary Material***

**Supplementary Tables**

**Supplementary Table 1** Location of sugarcane mills in three counties in the state of Alagoas used to collect plant material and the number of internodes of cultivated sugarcane varieties.

| **Mills** | **Location** | **Variety** | **Samples** | **Internodes number** |
| --- | --- | --- | --- | --- |
| Coruripe (County Coruripe) | Subarea 1  Lat: -10.040216 Long: -36.264507 | RB92 579  (5 stems) | 1 | 6 |
|  |  |  | 2 | 6 |
|  |  |  | 3 | 6 |
|  | Subarea 2  Lat: -10.035354 Long: -36.263864 | RB92 579  (5 stems) | 1 | 6 |
|  |  |  | 2 | 6 |
|  |  |  | 3 | 6 |
|  | Subarea 3  Lat: -10.100223 Long: - 36.274595 | RB92 579  (5 stems) | 1 | 6 |
|  |  |  | 2 | 6 |
|  |  |  | 3 | 6 |
| Triunfo (County Boca da Mata) | Subarea 1  Lat: -9.672355 Long: -36.097644 | RB86 7515  (5 stems) | 1 | 6 |
|  |  |  | 2 | 6 |
|  |  |  | 3 | 6 |
|  | Subarea 2  Lat: -9.664695 Long: -36.098515 | RB86 7515  (5 stems) | 1 | 6 |
|  |  |  | 2 | 6 |
|  |  |  | 3 | 6 |
|  | Subarea 3  Lat: -9.66439 Long: -36.100461 | RB86 7515  (5 stems) | 1 | 6 |
|  |  |  | 2 | 6 |
|  |  |  | 3 | 6 |
| Santo Antônio (County São Luís do Quitunde) | Subarea 1  Lat: -9.211982 Long: -35.584677 | Pé de Ferro  (5 stems) | 1 | 6 |
|  |  |  | 2 | 6 |
|  |  |  | 3 | 6 |
|  | Subarea 2  Lat: - 9.208459 Long: -35.584392 | RB95 1541  (5 stems) | 1 | 6 |
|  |  |  | 2 | 6 |
|  |  |  | 3 | 6 |
|  | Subárea 3  Lat: -9.201977 Long: -35.571106 | RB92 579  (5 stems) | 1 | 6 |
|  |  |  | 2 | 6 |
|  |  |  | 3 | 6 |

Supplementary Table 2 Phylogenetic analysis based on the 16S rRNA sequences of endophytic bacterial strains isolated from different niches of sugarcane plants and the digestive tract of the giant borer collected in sugarcane producing areas of the state of Alagoas (Continued).

| **Isolate** | **BLAST-N (NCBI)** | | | | |
| --- | --- | --- | --- | --- | --- |
|  | **Code** | **Mills** | **Identity (%)** | **GenBank Similarity** | **Access Number** |
| FORCN001 | CA 01 | Coruripe | 99.57 | *Bacillus licheniformis* | NR_118996.1 |
| FORCN002 | CA 02 | Coruripe | 99.71 | *Bacillus licheniformis* | [NR_118996.1](https://www.ncbi.nlm.nih.gov/nucleotide/NR_118996.1?report=genbank&log$=nucltop&blast_rank=1&RID=9UHX42KP014) |
| FORCN003 | CA 05 | Coruripe | 99.93 | *Bacillus megaterium* | [NR_112636.1](https://www.ncbi.nlm.nih.gov/nucleotide/NR_112636.1?report=genbank&log$=nucltop&blast_rank=1&RID=9UHZ4E6K015) |
| FORCN004 | CA 07 | Coruripe | 99.63 | *Bacillus aerius* | [NR_118439.1](https://www.ncbi.nlm.nih.gov/nucleotide/NR_118439.1?report=genbank&log$=nucltop&blast_rank=1&RID=9K4BTUH8014) |
| FORCN005 | CA 08 | Coruripe | 99.61 | *Bacillus thuringiensis* | [NR_114581.1](https://www.ncbi.nlm.nih.gov/nucleotide/NR_114581.1?report=genbank&log$=nucltop&blast_rank=1&RID=9UJ1ZC3W014) |
| FORCN006 | CA 27 | Coruripe | 99.50 | *Bacillus subtilis* | [NR_113265.1](https://www.ncbi.nlm.nih.gov/nucleotide/NR_113265.1?report=genbank&log$=nucltop&blast_rank=1&RID=9UJ4KHYC015) |
| FORCN007 | CA 35 | Triunfo | 99.86 | *Bacillus wiedmannii* | [NR_152692.1](https://www.ncbi.nlm.nih.gov/nucleotide/NR_152692.1?report=genbank&log$=nucltop&blast_rank=1&RID=9UJ6GEZ7014) |
| FORCN008 | CA 38 | Triunfo | 99.79 | *Bacillus proteolyticus* | [NR_157735.1](https://www.ncbi.nlm.nih.gov/nucleotide/NR_157735.1?report=genbank&log$=nucltop&blast_rank=1&RID=U2BGYRKA016) |
| FORCN009 | CA 42 | Triunfo | 99.06 | *Bacillus subterraneus* | [NR_104749.1](https://www.ncbi.nlm.nih.gov/nucleotide/NR_104749.1?report=genbank&log$=nucltop&blast_rank=1&RID=9UJ9ZNNH014) |
| FORCN010 | CA 44 | Triunfo | 99.57 | *Bacillus cereus* | [NR_074540.1](https://www.ncbi.nlm.nih.gov/nucleotide/NR_074540.1?report=genbank&log$=nucltop&blast_rank=1&RID=9UJBM3MP015) |
| FORCN011 | CA 48 | Triunfo | 99.86 | *Bacillus cereus* | [NR_074540.1](https://www.ncbi.nlm.nih.gov/nucleotide/NR_074540.1?report=genbank&log$=nucltop&blast_rank=1&RID=U2BUT02H013) |
| FORCN012 | CA 49 | Triunfo | 99.86 | *Bacillus cereus* | [NR_074540.1](https://www.ncbi.nlm.nih.gov/nucleotide/NR_074540.1?report=genbank&log$=nucltop&blast_rank=1&RID=9UJE8NPC015) |
| FORCN013 | CA 50 | Triunfo | 99.86 | *Bacillus australimaris* | [NR_148787.1](https://www.ncbi.nlm.nih.gov/nucleotide/NR_148787.1?report=genbank&log$=nucltop&blast_rank=1&RID=U2BWS71B013) |
| FORCN014 | CA 54 | Triunfo | 100.00 | *Bacillus cereus* | [NR_074540.1](https://www.ncbi.nlm.nih.gov/nucleotide/NR_074540.1?report=genbank&log$=nucltop&blast_rank=1&RID=9UJHDMPX015) |
| FORCN015 | CA 64 | Triunfo | 99.65 | *Terribacillus saccharophilus* | [NR_041356.1](https://www.ncbi.nlm.nih.gov/nucleotide/NR_041356.1?report=genbank&log$=nucltop&blast_rank=1&RID=9UMGXPMZ01R) |
| FORCN016 | CA 65 | Triunfo | 97.45 | *Paenibacillus vulneris* | [NR_117618.1](https://www.ncbi.nlm.nih.gov/nucleotide/NR_117618.1?report=genbank&log$=nucltop&blast_rank=1&RID=9UK0M5JT01R) |
| FORCN017 | CA 68 | Triunfo | 98.70 | *Bacillus aerius* | [NR_118439.1](https://www.ncbi.nlm.nih.gov/nucleotide/NR_118439.1?report=genbank&log$=nucltop&blast_rank=1&RID=9UK3RC5W01R) |
| FORCN018 | CA 70 | Triunfo | 99.79 | *Bacillus aerius* | [NR_118439.1](https://www.ncbi.nlm.nih.gov/nucleotide/NR_118439.1?report=genbank&log$=nucltop&blast_rank=1&RID=U2BYYNB0013) |
| FORCN019 | CA 73 | Santo Antônio | 99.50 | *Bacillus megaterium* | [NR_117473.1](https://www.ncbi.nlm.nih.gov/nucleotide/NR_117473.1?report=genbank&log$=nucltop&blast_rank=1&RID=9UK6EFP701R) |
| FORCN020 | CA 74 | Santo Antônio | 99.72 | *Bacillus aerius* | [NR_118439.1](https://www.ncbi.nlm.nih.gov/nucleotide/NR_118439.1?report=genbank&log$=nucltop&blast_rank=1&RID=9UK84UYA01R) |
| FORCN021 | CA 75 | Santo Antônio | 99.93 | *Bacillus aerius* | [NR_118439.1](https://www.ncbi.nlm.nih.gov/nucleotide/NR_118439.1?report=genbank&log$=nucltop&blast_rank=1&RID=9UKAKK4U01R) |
| FORCN022 | CA 77 | Santo Antônio | 99.46 | *Brevibacillus invocatus* | [NR_041836.1](https://www.ncbi.nlm.nih.gov/nucleotide/NR_041836.1?report=genbank&log$=nucltop&blast_rank=1&RID=U2C0WF9T016) |
| FORCN023 | CA 78 | Santo Antônio | 99.72 | *Bacillus aerius* | [NR_118439.1](https://www.ncbi.nlm.nih.gov/nucleotide/NR_118439.1?report=genbank&log$=nucltop&blast_rank=1&RID=U2CB3MM9013) |
| FORCN024 | CA 81 | Santo Antônio | 99.86 | *Bacillus aerius* | [NR_118439.1](https://www.ncbi.nlm.nih.gov/nucleotide/NR_118439.1?report=genbank&log$=nucltop&blast_rank=1&RID=9UYYWNZP014) |
| FORCN025 | CA 83 | Santo Antônio | 99.50 | *Bacillus megaterium* | [NR_117473.1](https://www.ncbi.nlm.nih.gov/nucleotide/NR_117473.1?report=genbank&log$=nucltop&blast_rank=1&RID=9UKG652H01R) |
| FORCN026 | CA 84 | Santo Antônio | 99.14 | *Bacillus safensis* | [NR_113945.1](https://www.ncbi.nlm.nih.gov/nucleotide/NR_113945.1?report=genbank&log$=nucltop&blast_rank=1&RID=9UKN0ZP601R) |
| FORCN027 | CA 85 | Santo Antônio | 99.93 | *Bacillus safensis* | [NR_113945.1](https://www.ncbi.nlm.nih.gov/nucleotide/NR_113945.1?report=genbank&log$=nucltop&blast_rank=1&RID=9UKPVM8H01R) |
| FORCN028 | CA 92 | Santo Antônio | 99.79 | *Bacillus megaterium* | [NR_117473.1](https://www.ncbi.nlm.nih.gov/nucleotide/NR_117473.1?report=genbank&log$=nucltop&blast_rank=1&RID=9UKTC9S001R) |
| FORCN029 | CA 96 | Santo Antônio | 99.86 | *Bacillus safensis* | [NR_113945.1](https://www.ncbi.nlm.nih.gov/nucleotide/NR_113945.1?report=genbank&log$=nucltop&blast_rank=1&RID=UWBU24TP015) |
| FORCN030 | CA 97 | Santo Antônio | 99.93 | *Bacillus megaterium* | [NR_112636.1](https://www.ncbi.nlm.nih.gov/nucleotide/NR_112636.1?report=genbank&log$=nucltop&blast_rank=1&RID=9UKV6TX901R) |
| FORCN031 | CA 101 | Santo Antônio | 99.93 | *Bacillus safensis* | [NR_113945.1](https://www.ncbi.nlm.nih.gov/nucleotide/NR_113945.1?report=genbank&log$=nucltop&blast_rank=1&RID=9UKZJEWM01R) |
| FORCN032 | CA 105 | Santo Antônio | 99.96 | *Paenibacillus illinoisensis* | [NR_113828.1](https://www.ncbi.nlm.nih.gov/nucleotide/NR_113828.1?report=genbank&log$=nucltop&blast_rank=1&RID=9UM27SH201R) |
| FORCN033 | CA 108 | Santo Antônio | 99.14 | *Bacillus licheniformis* | [NR_118996.1](https://www.ncbi.nlm.nih.gov/nucleotide/NR_118996.1?report=genbank&log$=nucltop&blast_rank=1&RID=9UM637EF01R) |
| FORCN034 | CA 109 | Santo Antônio | 98.77 | *Paenibacillus illinoisensis* | [NR_113828.1](https://www.ncbi.nlm.nih.gov/nucleotide/NR_113828.1?report=genbank&log$=nucltop&blast_rank=1&RID=9UR3ESRP01R) |
| FORCN035 | CA 110 | Santo Antônio | 99.93 | *Bacillus megaterium* | [NR_112636.1](https://www.ncbi.nlm.nih.gov/nucleotide/NR_112636.1?report=genbank&log$=nucltop&blast_rank=1&RID=U2CN6EZ6016) |
| FORCN036 | CA 113 | Santo Antônio | 99.63 | *Bacillus megaterium* | [NR_117473.1](https://www.ncbi.nlm.nih.gov/nucleotide/NR_117473.1?report=genbank&log$=nucltop&blast_rank=1&RID=9URBV3UK01R) |
| FORCN037 | CA 114 | Santo Antônio | 98.64 | *Paenibacillus illinoisensis* | [NR_113828.1](https://www.ncbi.nlm.nih.gov/nucleotide/NR_113828.1?report=genbank&log$=nucltop&blast_rank=1&RID=9UREA86S01R) |
| FORCN038 | CA 117 | Santo Antônio | 98.95 | *Paenibacillus illinoisensis* | [NR_113828.1](https://www.ncbi.nlm.nih.gov/nucleotide/NR_113828.1?report=genbank&log$=nucltop&blast_rank=1&RID=U2CSYW33016) |
| FORCN039 | CA 120 | Santo Antônio | 99.95 | *Paenibacillus illinoisensis* | [NR_113828.1](https://www.ncbi.nlm.nih.gov/nucleotide/NR_113828.1?report=genbank&log$=nucltop&blast_rank=1&RID=UJCV9KST015) |
| FORCN040 | CA 132 | Santo Antônio | 99.86 | *Bacillus albus* | [NR_157729.1](https://www.ncbi.nlm.nih.gov/nucleotide/NR_157729.1?report=genbank&log$=nucltop&blast_rank=1&RID=PPTV9TD3014) |
| FORCN041 | CA 140 | Santo Antônio | 99.86 | *Bacillus albus* | [NR_157729.1](https://www.ncbi.nlm.nih.gov/nucleotide/NR_157729.1?report=genbank&log$=nucltop&blast_rank=1&RID=PPTV9TD3014) |
| FORCN042 | CA 153 | Santo Antônio | 99.26 | *Terribacillus saccharophilus* | [NR_041356.1](https://www.ncbi.nlm.nih.gov/nucleotide/NR_041356.1?report=genbank&log$=nucltop&blast_rank=1&RID=9URK55P001R) |
| FORCN043 | CA 159 | Santo Antônio | 99.34 | *Bacillus drentensis* | [NR_114085.1](https://www.ncbi.nlm.nih.gov/nucleotide/NR_114085.1?report=genbank&log$=nucltop&blast_rank=1&RID=9URMSP7701R) |
| FORCN044 | CA 191 | Santo Antônio | 99.79 | *Bacillus megaterium* | [NR_117473.1](https://www.ncbi.nlm.nih.gov/nucleotide/NR_117473.1?report=genbank&log$=nucltop&blast_rank=1&RID=9URRE49401R) |
| FORCN045 | CA 193 | Santo Antônio | 99.71 | *Bacillus licheniformis* | [NR_118996.1](https://www.ncbi.nlm.nih.gov/nucleotide/NR_118996.1?report=genbank&log$=nucltop&blast_rank=1&RID=9UZ0NWZX014) |
| FORCN046 | CA 219 | Santo Antônio | 99.86 | *Bacillus safensis* | [NR_113945.1](https://www.ncbi.nlm.nih.gov/nucleotide/NR_113945.1?report=genbank&log$=nucltop&blast_rank=1&RID=9UZ2C4NT015) |
| FORCN047 | CB 01 | Santo Antônio | 100.00 | *Bacillus cereus* | [NR_074540.1](https://www.ncbi.nlm.nih.gov/nucleotide/NR_074540.1?report=genbank&log$=nucltop&blast_rank=1&RID=UJD3DR2Z014) |
| FORCN048 | CB 05 | Santo Antônio | 100.00 | *Bacillus cereus* | [NR_074540.1](https://www.ncbi.nlm.nih.gov/nucleotide/NR_074540.1?report=genbank&log$=nucltop&blast_rank=1&RID=9UGSSZ8F014) |
| FORCN049 | CB 08 | Santo Antônio | 100.00 | *Bacillus cereus* | [NR_074540.1](https://www.ncbi.nlm.nih.gov/nucleotide/NR_074540.1?report=genbank&log$=nucltop&blast_rank=1&RID=9UH7N2D9014) |
| FORCN050 | CB 09 | Triunfo | 99.93 | *Bacillus australimaris* | [NR_148787.1](https://www.ncbi.nlm.nih.gov/nucleotide/NR_148787.1?report=genbank&log$=nucltop&blast_rank=1&RID=9UH5889F015) |
| FORCN051 | CB 12 | Triunfo | 99.93 | *Bacillus australimaris* | [NR_148787.1](https://www.ncbi.nlm.nih.gov/nucleotide/NR_148787.1?report=genbank&log$=nucltop&blast_rank=1&RID=9NA1XZE1014) |
| FORCN052 | CB 15 | Triunfo | 99.93 | *Bacillus safensis* | [NR_041794.1](https://www.ncbi.nlm.nih.gov/nucleotide/NR_041794.1?report=genbank&log$=nucltop&blast_rank=1&RID=9N9X57VN015) |

**Supplementary Table 2** Continuation.

| FORCN053 | CB 16 | Triunfo | 99.86 | *Bacillus safensis* | [NR_041794.1](https://www.ncbi.nlm.nih.gov/nucleotide/NR_041794.1?report=genbank&log$=nucltop&blast_rank=1&RID=UJD67PBH014) |
| --- | --- | --- | --- | --- | --- |
| FORCN054 | CB 17 | Triunfo | 99.84 | *Bacillus safensis* | [NR_113945.1](https://www.ncbi.nlm.nih.gov/nucleotide/NR_113945.1?report=genbank&log$=nucltop&blast_rank=1&RID=9UMN9XP801R) |
| FORCN055 | CB 18 | Triunfo | 100.00 | *Bacillus safensis* | [NR_041794.1](https://www.ncbi.nlm.nih.gov/nucleotide/NR_041794.1?report=genbank&log$=nucltop&blast_rank=1&RID=9UG5NA44014) |
| FORCN056 | CB 19 | Triunfo | 99.78 | *Bacillus australimaris* | [NR_148787.1](https://www.ncbi.nlm.nih.gov/nucleotide/NR_148787.1?report=genbank&log$=nucltop&blast_rank=1&RID=9UMM088701R) |
| FORCN057 | CB 20 | Triunfo | 99.64 | *Bacillus australimaris* | [NR_148787.1](https://www.ncbi.nlm.nih.gov/nucleotide/NR_148787.1?report=genbank&log$=nucltop&blast_rank=1&RID=9UZE1SBZ015) |
| FORCN058 | CB 24 | Triunfo | 99.86 | *Bacillus safensis* | [NR_113945.1](https://www.ncbi.nlm.nih.gov/nucleotide/NR_113945.1?report=genbank&log$=nucltop&blast_rank=1&RID=9UYAZY9W015) |
| FORCN059 | CB 32 | Triunfo | 99.71 | *Bacillus safensis* | [NR_113945.1](https://www.ncbi.nlm.nih.gov/nucleotide/NR_113945.1?report=genbank&log$=nucltop&blast_rank=1&RID=9UYH62G7015) |
| FORCN060 | CB 35 | Triunfo | 99.50 | *Paenibacillus illinoisensis* | [NR_113828.1](https://www.ncbi.nlm.nih.gov/nucleotide/NR_113828.1?report=genbank&log$=nucltop&blast_rank=1&RID=9UYDDE60014) |
| FORCN061 | CB 36 | Triunfo | 99.86 | *Bacillus safensis* | [NR_113945.1](https://www.ncbi.nlm.nih.gov/nucleotide/NR_113945.1?report=genbank&log$=nucltop&blast_rank=1&RID=9UYH62G7015) |
| FORCN062 | CB 45 | Triunfo | 99.93 | *Bacillus australimaris* | [NR_148787.1](https://www.ncbi.nlm.nih.gov/nucleotide/NR_148787.1?report=genbank&log$=nucltop&blast_rank=1&RID=9UYNR9X5014) |
| FORCN063 | CB 51 | Triunfo | 99.64 | *Paenibacillus barcinonensis* | [NR_042272.1](https://www.ncbi.nlm.nih.gov/nucleotide/NR_042272.1?report=genbank&log$=nucltop&blast_rank=1&RID=9UYRVFJD014) |
| FORCN064 | CB 52 | Triunfo | 99.85 | *Bacillus safensis* | [NR_113945.1](https://www.ncbi.nlm.nih.gov/nucleotide/NR_113945.1?report=genbank&log$=nucltop&blast_rank=1&RID=9UYTNTYF014) |
| FORCN065 | CB 56 | Triunfo | 99.85 | *Bacillus safensis* | [NR_113945.1](https://www.ncbi.nlm.nih.gov/nucleotide/NR_113945.1?report=genbank&log$=nucltop&blast_rank=1&RID=9UYTNTYF014) |
| FORCN066 | CR 01 | Coruripe | 99.93 | *Bacillus cereus* | [NR_074540.1](https://www.ncbi.nlm.nih.gov/nucleotide/NR_074540.1?report=genbank&log$=nucltop&blast_rank=1&RID=9UZ479HT015) |
| FORCN067 | CR 02 | Coruripe | 100.00 | *Bacillus cereus* | [NR_074540.1](https://www.ncbi.nlm.nih.gov/nucleotide/NR_074540.1?report=genbank&log$=nucltop&blast_rank=1&RID=UJDBAE5R014) |
| FORCN068 | CR 07 | Coruripe | 99.70 | *Bacillus cereus* | [NR_074540.1](https://www.ncbi.nlm.nih.gov/nucleotide/NR_074540.1?report=genbank&log$=nucltop&blast_rank=1&RID=9UZ6NVUM015) |
| FORCN069 | CR 08 | Coruripe | 99.85 | *Bacillus cereus* | [NR_074540.1](https://www.ncbi.nlm.nih.gov/nucleotide/NR_074540.1?report=genbank&log$=nucltop&blast_rank=1&RID=9UZ6NVUM015) |
| FORCN070 | CR 12 | Coruripe | 99.40 | *Bacillus pacificus* | [NR_157733.1](https://www.ncbi.nlm.nih.gov/nucleotide/NR_157733.1?report=genbank&log$=nucltop&blast_rank=1&RID=UJDKRWMR015) |
| FORCN071 | CR 21 | Coruripe | 99.86 | *Bacillus cereus* | [NR_074540.1](https://www.ncbi.nlm.nih.gov/nucleotide/NR_074540.1?report=genbank&log$=nucltop&blast_rank=1&RID=9UZN2P2A015) |
| FORCN072 | CR 22 | Coruripe | 99.48 | *Bacillus safensis* | [NR_113945.1](https://www.ncbi.nlm.nih.gov/nucleotide/NR_113945.1?report=genbank&log$=nucltop&blast_rank=1&RID=9UZ9UD3M015) |
| FORCN073 | CR 24 | Coruripe | 100.00 | *Bacillus tequilensis* | [NR_104919.1](https://www.ncbi.nlm.nih.gov/nucleotide/NR_104919.1?report=genbank&log$=nucltop&blast_rank=1&RID=9UMSTASE01R) |
| FORCN074 | CR 25 | Coruripe | 99.86 | *Bacillus subtilis* | [NR_112629.1](https://www.ncbi.nlm.nih.gov/nucleotide/NR_112629.1?report=genbank&log$=nucltop&blast_rank=1&RID=UJDPCUE9015) |
| FORCN075 | CR 26 | Coruripe | 99.03 | *Bacillus pacificus* | [NR_157733.1](https://www.ncbi.nlm.nih.gov/nucleotide/NR_157733.1?report=genbank&log$=nucltop&blast_rank=1&RID=UJDV7D7Z015) |
| FORCN076 | CR 27 | Coruripe | 98.59 | *Bacillus pacificus* | [NR_157733.1](https://www.ncbi.nlm.nih.gov/nucleotide/NR_157733.1?report=genbank&log$=nucltop&blast_rank=1&RID=UJE2SMFJ015) |
| FORCN077 | CR 28 | Coruripe | 99.49 | *Bacillus amyloliquefaciens* | [NR_117946.1](https://www.ncbi.nlm.nih.gov/nucleotide/NR_117946.1?report=genbank&log$=nucltop&blast_rank=1&RID=9UZFH8KW014) |
| FORCN078 | CR 29 | Coruripe | 99.57 | *Bacillus amyloliquefaciens* | [NR_117946.1](https://www.ncbi.nlm.nih.gov/nucleotide/NR_117946.1?report=genbank&log$=nucltop&blast_rank=1&RID=9UZHVS79014) |
| FORCN079 | CR 30 | Coruripe | 99.62 | *Bacillus amyloliquefaciens* | [NR_117946.1](https://www.ncbi.nlm.nih.gov/nucleotide/NR_117946.1?report=genbank&log$=nucltop&blast_rank=1&RID=9UZHVS79014) |
| FORCN080 | CR 31 | Coruripe | 99.77 | *Bacillus amyloliquefaciens* | [NR_117946.1](https://www.ncbi.nlm.nih.gov/nucleotide/NR_117946.1?report=genbank&log$=nucltop&blast_rank=1&RID=9UZSVA0W015) |
| FORCN081 | CR 32 | Coruripe | 99.86 | *Bacillus subtilis* | [NR_113265.1](https://www.ncbi.nlm.nih.gov/nucleotide/NR_113265.1?report=genbank&log$=nucltop&blast_rank=1&RID=UJE512ES014) |
| FORCN082 | CR 33 | Coruripe | 99.85 | *Bacillus tequilensis* | [NR_104919.1](https://www.ncbi.nlm.nih.gov/nucleotide/NR_104919.1?report=genbank&log$=nucltop&blast_rank=1&RID=9UMXMMYE01R) |
| FORCN083 | CR 34 | Coruripe | 99.86 | *Bacillus tequilensis* | [NR_104919.1](https://www.ncbi.nlm.nih.gov/nucleotide/NR_104919.1?report=genbank&log$=nucltop&blast_rank=1&RID=9UNK1GA601R) |
| FORCN084* | CR35 | Coruripe | - | - | - |
| FORCN085 | CR 38 | Coruripe | 99.93 | *Bacillus megaterium* | [NR_117473.1](https://www.ncbi.nlm.nih.gov/nucleotide/NR_117473.1?report=genbank&log$=nucltop&blast_rank=1&RID=9UNNN0UZ01R) |
| FORCN086 | CR 43 | Coruripe | 99.86 | *Bacillus megaterium* | [NR_117473.1](https://www.ncbi.nlm.nih.gov/nucleotide/NR_117473.1?report=genbank&log$=nucltop&blast_rank=1&RID=9UNRWCV201R) |
| FORCN087 | CR 45 | Coruripe | 98.70 | *Bacillus pacificus* | [NR_157733.1](https://www.ncbi.nlm.nih.gov/nucleotide/NR_157733.1?report=genbank&log$=nucltop&blast_rank=1&RID=UJEA6AZ0015) |
| FORCN088 | CR 46 | Coruripe | 99.72 | *Bacillus amyloliquefaciens* | [NR_041455.1](https://www.ncbi.nlm.nih.gov/nucleotide/NR_041455.1?report=genbank&log$=nucltop&blast_rank=1&RID=UJEGP20R014) |
| FORCN089 | CR 49 | Coruripe | 99.96 | *Bacillus pacificus* | [NR_157733.1](https://www.ncbi.nlm.nih.gov/nucleotide/NR_157733.1?report=genbank&log$=nucltop&blast_rank=1&RID=UJEK40MP014) |
| FORCN090 | CR 50 | Coruripe | 99.16 | *Bacillus pacificus* | [NR_157733.1](https://www.ncbi.nlm.nih.gov/nucleotide/NR_157733.1?report=genbank&log$=nucltop&blast_rank=1&RID=UJER0NCM014) |
| FORCN091 | CR 51 | Coruripe | 98.92 | *Bacillus pacificus* | [NR_157733.1](https://www.ncbi.nlm.nih.gov/nucleotide/NR_157733.1?report=genbank&log$=nucltop&blast_rank=1&RID=UJEY2AZD015) |
| FORCN092 | CR 52 | Coruripe | 98.97 | *Bacillus pacificus* | [NR_157733.1](https://www.ncbi.nlm.nih.gov/nucleotide/NR_157733.1?report=genbank&log$=nucltop&blast_rank=1&RID=UJF0K4JU014) |
| FORCN093 | CR 53 | Coruripe | 98.32 | *Bacillus pacificus* | [NR_157733.1](https://www.ncbi.nlm.nih.gov/nucleotide/NR_157733.1?report=genbank&log$=nucltop&blast_rank=1&RID=UJF7DWEA015) |
| FORCN094 | CR 54 | Coruripe | 99.61 | *Bacillus megaterium* | [NR_117473.1](https://www.ncbi.nlm.nih.gov/nucleotide/NR_117473.1?report=genbank&log$=nucltop&blast_rank=1&RID=9URSZJ0S01R) |
| FORCN095 | CR 55 | Coruripe | 99.20 | *Bacillus megaterium* | [NR_117473.1](https://www.ncbi.nlm.nih.gov/nucleotide/NR_117473.1?report=genbank&log$=nucltop&blast_rank=1&RID=9UY7PKYF015) |
| FORCN096 | CR 59 | Coruripe | 98.59 | *Bacillus pacificus* | [NR_157733.1](https://www.ncbi.nlm.nih.gov/nucleotide/NR_157733.1?report=genbank&log$=nucltop&blast_rank=1&RID=UTSVXAPA01R) |
| FORCN097* | CR68 | Triunfo | - | - | - |
| FORCN098 | CR 69 | Triunfo | 99.72 | *Bacillus megaterium* | [NR_117473.1](https://www.ncbi.nlm.nih.gov/nucleotide/NR_117473.1?report=genbank&log$=nucltop&blast_rank=1&RID=UTTABUDF01R) |
| FORCN099 | CR 70 | Triunfo | 97.81 | *Paenibacillus alvei* | [NR_113577.1](https://www.ncbi.nlm.nih.gov/nucleotide/NR_113577.1?report=genbank&log$=nucltop&blast_rank=1&RID=UTTC012K01R) |
| FORCN100 | CR 76 | Triunfo | 99.57 | *Bacillus amyloliquefaciens* | [NR_117946.1](https://www.ncbi.nlm.nih.gov/nucleotide/NR_117946.1?report=genbank&log$=nucltop&blast_rank=1&RID=9UGR6HEE015) |
| FORCN101 | CR 77 | Triunfo | 99.79 | *Bacillus amyloliquefaciens* | [NR_041455.1](https://www.ncbi.nlm.nih.gov/nucleotide/NR_041455.1?report=genbank&log$=nucltop&blast_rank=1&RID=9N938BRK015) |
| FORCN102 | CR 79 | Triunfo | 99.48 | *Bacillus amyloliquefaciens* | [NR_117946.1](https://www.ncbi.nlm.nih.gov/nucleotide/NR_117946.1?report=genbank&log$=nucltop&blast_rank=1&RID=9UPUJJGU01R) |
| FORCN103 | CR 80 | Triunfo | 99.17 | *Bacillus cereus* | [NR_074540.1](https://www.ncbi.nlm.nih.gov/nucleotide/NR_074540.1?report=genbank&log$=nucltop&blast_rank=1&RID=9UR00PSZ01R) |
| FORCN104 | CR 81 | Triunfo | 100.00 | *Bacillus proteolyticus* | [NR_157735.1](https://www.ncbi.nlm.nih.gov/nucleotide/NR_157735.1?report=genbank&log$=nucltop&blast_rank=1&RID=MWM6YT2D016) |
| FORCN105 | CR 82 | Triunfo | 99.79 | *Lysinibacillus fusiformis* | [NR_112569.1](https://www.ncbi.nlm.nih.gov/nucleotide/NR_112569.1?report=genbank&log$=nucltop&blast_rank=1&RID=9UHR78G6014) |
| FORCN106 | CR 91 | Triunfo | 99.86 | *Bacillus subterraneus* | [NR_104749.1](https://www.ncbi.nlm.nih.gov/nucleotide/NR_104749.1?report=genbank&log$=nucltop&blast_rank=1&RID=9UG382Y3014) |
| FORCN107 | CR 95 | Triunfo | 99.72 | *Bacillus megaterium* | [NR_112636.1](https://www.ncbi.nlm.nih.gov/nucleotide/NR_112636.1?report=genbank&log$=nucltop&blast_rank=1&RID=UTTFKUGK01R) |
| FORCN108 | CR 98 | Triunfo | 99.93 | *Bacillus megaterium* | [NR_117473.1](https://www.ncbi.nlm.nih.gov/nucleotide/NR_117473.1?report=genbank&log$=nucltop&blast_rank=1&RID=UTTH5VXG01R) |
| FORCN109 | CR 107 | Triunfo | 99.36 | *Bacillus subterraneus* | [NR_104749.1](https://www.ncbi.nlm.nih.gov/nucleotide/NR_104749.1?report=genbank&log$=nucltop&blast_rank=1&RID=9N9638UU01R) |
| FORCN110 | CR 126 | Santo Antônio | 99.86 | *Bacillus amyloliquefaciens* | [NR_041455.1](https://www.ncbi.nlm.nih.gov/nucleotide/NR_041455.1?report=genbank&log$=nucltop&blast_rank=1&RID=9N7CUGJN014) |

**Supplementary Table 2** Continuation.

| FORCN111 | CR 129 | Santo Antônio | 99.72 | *Bacillus amyloliquefaciens* | [NR_041455.1](https://www.ncbi.nlm.nih.gov/nucleotide/NR_041455.1?report=genbank&log$=nucltop&blast_rank=1&RID=UTTJYMBW01R) |
| --- | --- | --- | --- | --- | --- |
| FORCN112 | CR 132 | Santo Antônio | 99.44 | *Fictibacillus barbaricus* | [NR_028967.1](https://www.ncbi.nlm.nih.gov/nucleotide/NR_028967.1?report=genbank&log$=nucltop&blast_rank=1&RID=UTTP10GH01R) |
| FORCN113 | CR 136 | Santo Antônio | 99.65 | *Bacillus amyloliquefaciens* | [NR_041455.1](https://www.ncbi.nlm.nih.gov/nucleotide/NR_041455.1?report=genbank&log$=nucltop&blast_rank=1&RID=UTU27Z7701R) |
| FORCN114 | CR 157 | Santo Antônio | 100.00 | *Bacillus flexus* | [NR_113800.1](https://www.ncbi.nlm.nih.gov/nucleotide/NR_113800.1?report=genbank&log$=nucltop&blast_rank=1&RID=9N78F58H014) |
| FORCN115 | CR 158 | Santo Antônio | 99.86 | *Bacillus flexus* | [NR_113800.1](https://www.ncbi.nlm.nih.gov/nucleotide/NR_113800.1?report=genbank&log$=nucltop&blast_rank=1&RID=9N991ET4015) |
| FORCN116 | CC 01 | Coruripe | 99.55 | *Bacillus velezensis* | [NR_116240.1](https://www.ncbi.nlm.nih.gov/nucleotide/NR_116240.1?report=genbank&log$=nucltop&blast_rank=1&RID=MWMEFM7A014) |
| FORCN117 | CC 03 | Coruripe | 99.57 | *Bacillus velezensis* | [NR_116240.1](https://www.ncbi.nlm.nih.gov/nucleotide/NR_116240.1?report=genbank&log$=nucltop&blast_rank=1&RID=MWMJ3D9G016) |
| FORCN118 | CC 06 | Coruripe | 99.65 | *Bacillus oleronius* | [NR_043325.1](https://www.ncbi.nlm.nih.gov/nucleotide/NR_043325.1?report=genbank&log$=nucltop&blast_rank=1&RID=UTU99DY301R) |
| FORCN119 | CC 07 | Coruripe | 100.00 | *Bacillus megaterium* | [NR_112636.1](https://www.ncbi.nlm.nih.gov/nucleotide/NR_112636.1?report=genbank&log$=nucltop&blast_rank=1&RID=UTUBEWYG01R) |
| FORCN120 | CC 10 | Coruripe | 99.77 | *Bacillus oleronius* | [NR_043325.1](https://www.ncbi.nlm.nih.gov/nucleotide/NR_043325.1?report=genbank&log$=nucltop&blast_rank=1&RID=9UNXSW0A01R) |
| FORCN121 | CC 17 | Coruripe | 99.79 | *Bacillus oleronius* | [NR_043325.1](https://www.ncbi.nlm.nih.gov/nucleotide/NR_043325.1?report=genbank&log$=nucltop&blast_rank=1&RID=9UP22VTM01R) |
| FORCN122 | CC 19 | Coruripe | 99.06 | *Bacillus oleronius* | [NR_043325.1](https://www.ncbi.nlm.nih.gov/nucleotide/NR_043325.1?report=genbank&log$=nucltop&blast_rank=1&RID=9UPHWZYM01R) |
| FORCN123 | CC 24 | Coruripe | 99.93 | *Bacillus circulans* | [NR_112632.1](https://www.ncbi.nlm.nih.gov/nucleotide/NR_112632.1?report=genbank&log$=nucltop&blast_rank=1&RID=UTUCWPEC01R) |
| FORCN124 | CC 28 | Coruripe | 99.78 | *Bacillus velezensis* | [NR_075005.2](https://www.ncbi.nlm.nih.gov/nucleotide/NR_075005.2?report=genbank&log$=nucltop&blast_rank=1&RID=9UPKW48N01R) |
| FORCN125 | CC 35 | Coruripe | 99.41 | *Bacillus safensis* | [NR_113945.1](https://www.ncbi.nlm.nih.gov/nucleotide/NR_113945.1?report=genbank&log$=nucltop&blast_rank=1&RID=9UPN808D01R) |
| FORCN126 | CC 36 | Coruripe | 99.93 | *Bacillus safensis* | [NR_113945.1](https://www.ncbi.nlm.nih.gov/nucleotide/NR_113945.1?report=genbank&log$=nucltop&blast_rank=1&RID=9UPB3JRX01R) |
| FORCN127 | CC 64 | Coruripe | 99.78 | *Bacillus megaterium* | [NR_116873.1](https://www.ncbi.nlm.nih.gov/nucleotide/NR_116873.1?report=genbank&log$=nucltop&blast_rank=1&RID=9UP438M701R) |
| FORCN128 | CC 67 | Coruripe | 94.07 | *Lysinibacillus cresolivorans* | [NR_145635.1](https://www.ncbi.nlm.nih.gov/nucleotide/NR_145635.1?report=genbank&log$=nucltop&blast_rank=1&RID=UW9HP42J015) |
| FORCN129 | CC 96 | Coruripe | 99.64 | *Bacillus oleronius* | [NR_043325.1](https://www.ncbi.nlm.nih.gov/nucleotide/NR_043325.1?report=genbank&log$=nucltop&blast_rank=1&RID=9UP820NC01R) |
| FORCN130 | CC 100 | Coruripe | 99.86 | *Bacillus megaterium* | [NR_112636.1](https://www.ncbi.nlm.nih.gov/nucleotide/NR_112636.1?report=genbank&log$=nucltop&blast_rank=1&RID=UW9P9DVK015) |
| FORCN131 | CC 108 | Coruripe | 100.00 | *Bacillus cereus* | [NR_074540.1](https://www.ncbi.nlm.nih.gov/nucleotide/NR_074540.1?report=genbank&log$=nucltop&blast_rank=1&RID=9UPFT06F01R) |
| FORCN132 | CC 110 | Coruripe | 99.72 | *Bacillus oleronius* | [NR_043325.1](https://www.ncbi.nlm.nih.gov/nucleotide/NR_043325.1?report=genbank&log$=nucltop&blast_rank=1&RID=UW9S7RYU014) |
| FORCN133 | CC 127 | Coruripe | 99.93 | *Bacillus circulans* | [NR_112632.1](https://www.ncbi.nlm.nih.gov/nucleotide/NR_112632.1?report=genbank&log$=nucltop&blast_rank=1&RID=UW9TS5PT014) |
| FORCN134 | CC 141 | Coruripe | 99.70 | *Bacillus albus* | [NR_157729.1](https://www.ncbi.nlm.nih.gov/nucleotide/NR_157729.1?report=genbank&log$=nucltop&blast_rank=1&RID=PR1YH7JX016) |
| FORCN135 | CC 144 | Coruripe | 99.93 | *Bacillus albus* | [NR_157729.1](https://www.ncbi.nlm.nih.gov/nucleotide/NR_157729.1?report=genbank&log$=nucltop&blast_rank=1&RID=PR1YH7JX016) |
| FORCN136 | CC 146 | Coruripe | 99.44 | *Bacillus oleronius* | [NR_043325.1](https://www.ncbi.nlm.nih.gov/nucleotide/NR_043325.1?report=genbank&log$=nucltop&blast_rank=1&RID=9N8V6MJW014) |
| FORCN137 | CC 150 | Coruripe | 99.58 | *Bacillus oleronius* | [NR_043325.1](https://www.ncbi.nlm.nih.gov/nucleotide/NR_043325.1?report=genbank&log$=nucltop&blast_rank=1&RID=9N7G74D7015) |
| FORCN138 | CC 153 | Coruripe | 99.93 | *Bacillus albus* | [NR_157729.1](https://www.ncbi.nlm.nih.gov/nucleotide/NR_157729.1?report=genbank&log$=nucltop&blast_rank=1&RID=PR1YH7JX016) |
| FORCN139 | CC 157 | Coruripe | 100.00 | *Bacillus albus* | [NR_157736.1](https://www.ncbi.nlm.nih.gov/nucleotide/NR_157736.1?report=genbank&log$=nucltop&blast_rank=1&RID=MWMPGP3U014) |
| FORCN140 | CC 173 | Triunfo | 97.60 | *Bacillus circulans* | [NR_112632.1](https://www.ncbi.nlm.nih.gov/nucleotide/NR_112632.1?report=genbank&log$=nucltop&blast_rank=1&RID=UW9ZB2MU014) |
| FORCN141 | CC 179 | Triunfo | 100.00 | *Bacillus circulans* | [NR_112632.1](https://www.ncbi.nlm.nih.gov/nucleotide/NR_112632.1?report=genbank&log$=nucltop&blast_rank=1&RID=9UH2Y0HH014) |
| FORCN142 | CC 180 | Triunfo | 99.85 | *Bacillus circulans* | [NR_112632.1](https://www.ncbi.nlm.nih.gov/nucleotide/NR_112632.1?report=genbank&log$=nucltop&blast_rank=1&RID=9UGGHH9H015) |
| FORCN143 | CC 189 | Triunfo | 99.86 | *Bacillus circulans* | [NR_112632.1](https://www.ncbi.nlm.nih.gov/nucleotide/NR_112632.1?report=genbank&log$=nucltop&blast_rank=1&RID=9UHJ4UNP014) |
| FORCN144 | CC 192 | Triunfo | 97.64 | *Bacillus circulans* | [NR_112632.1](https://www.ncbi.nlm.nih.gov/nucleotide/NR_112632.1?report=genbank&log$=nucltop&blast_rank=1&RID=UWA0YS6G015) |
| FORCN145 | CC 193 | Triunfo | 99.71 | *Lysinibacillus macroides* | [NR_114920.1](https://www.ncbi.nlm.nih.gov/nucleotide/NR_114920.1?report=genbank&log$=nucltop&blast_rank=1&RID=9UGZR8T2015) |
| FORCN146 | CC 201 | Triunfo | 99.86 | *Bacillus circulans* | [NR_112632.1](https://www.ncbi.nlm.nih.gov/nucleotide/NR_112632.1?report=genbank&log$=nucltop&blast_rank=1&RID=UWBP2ZE3015) |
| FORCN147 | CC 202 | Triunfo | 99.01 | *Bacillus nealsonii* | [NR_044546.1](https://www.ncbi.nlm.nih.gov/nucleotide/NR_044546.1?report=genbank&log$=nucltop&blast_rank=1&RID=9N8KRESV01R) |
| FORCN148 | CC 208 | Triunfo | 99.58 | *Bacillus oleronius* | [NR_043325.1](https://www.ncbi.nlm.nih.gov/nucleotide/NR_043325.1?report=genbank&log$=nucltop&blast_rank=1&RID=9UGBGKF0015) |
| FORCN149 | CC 210 | Triunfo | 99.64 | *Bacillus oleronius* | [NR_043325.1](https://www.ncbi.nlm.nih.gov/nucleotide/NR_043325.1?report=genbank&log$=nucltop&blast_rank=1&RID=9UGXWG6H014) |
| FORCN150 | CC 212 | Triunfo | 99.71 | *Bacillus licheniformis* | [NR_118996.1](https://www.ncbi.nlm.nih.gov/nucleotide/NR_118996.1?report=genbank&log$=nucltop&blast_rank=1&RID=9UHECKAJ015) |
| FORCN151 | CC 214 | Triunfo | 99.65 | *Bacillus kochii* | [NR_117050.1](https://www.ncbi.nlm.nih.gov/nucleotide/NR_117050.1?report=genbank&log$=nucltop&blast_rank=1&RID=9N6ZB73C015) |
| FORCN152 | CC 215 | Triunfo | 99.57 | *Lysinibacillus macroides* | [NR_114920.1](https://www.ncbi.nlm.nih.gov/nucleotide/NR_114920.1?report=genbank&log$=nucltop&blast_rank=1&RID=9N8SPZTZ015) |
| FORCN153 | CC 220 | Triunfo | 99.86 | *Bacillus oleronius* | [NR_043325.1](https://www.ncbi.nlm.nih.gov/nucleotide/NR_043325.1?report=genbank&log$=nucltop&blast_rank=1&RID=9UGVVAAF015) |
| FORCN154 | CC 222 | Triunfo | 99.64 | *Bacillus oleronius* | [NR_043325.1](https://www.ncbi.nlm.nih.gov/nucleotide/NR_043325.1?report=genbank&log$=nucltop&blast_rank=1&RID=9UHGEG0A014) |
| FORCN155* | CC224 | Triunfo | - | - | - |
| FORCN156 | CC 233 | Triunfo | 99.58 | *Lysinibacillus macroides* | [NR_114920.1](https://www.ncbi.nlm.nih.gov/nucleotide/NR_114920.1?report=genbank&log$=nucltop&blast_rank=1&RID=9N6NRW5U014) |
| FORCN157 | CC 238 | Triunfo | 98.81 | *Lysinibacillus macroides* | [NR_114920.1](https://www.ncbi.nlm.nih.gov/nucleotide/NR_114920.1?report=genbank&log$=nucltop&blast_rank=1&RID=9UFWKCDV015) |
| FORCN158 | CC 243 | Triunfo | 99.48 | *Bacillus oleronius* | [NR_043325.1](https://www.ncbi.nlm.nih.gov/nucleotide/NR_043325.1?report=genbank&log$=nucltop&blast_rank=1&RID=9UG05834014) |
| FORCN159 | CC 250 | Triunfo | 99.64 | *Bacillus oleronius* | [NR_043325.1](https://www.ncbi.nlm.nih.gov/nucleotide/NR_043325.1?report=genbank&log$=nucltop&blast_rank=1&RID=9UGE4CHF014) |
| FORCN160 | CC 337 | Santo Antônio | 99.79 | *Bacillus velezensis* | [NR_075005.2](https://www.ncbi.nlm.nih.gov/nucleotide/NR_075005.2?report=genbank&log$=nucltop&blast_rank=1&RID=9N8Y5FD6015) |
| FORCN161* | CC355 | Santo Antônio | - | - | - |

CA – isolated from apoplasto fluid of sugarcane; CB – isolated from digestive tract of giant borer larvae; CR – isolated from roots of sugarcane; CC – isolated from central region of internodes of sugarcane.

*Isolates not sequenced, as they did not amplify.

Supplementary Table 3 Phylogenetic analysis based on the 16S rRNA sequences of bacterial strains isolated from the digestive tract of the giant borer collected in sugarcane producing areas in the state of Alagoas.

| **Isolate** | **Code** | **Mills** | **BLAST-N (NCBI)** | | |
| --- | --- | --- | --- | --- | --- |
|  |  |  | **GenBank Similarity** | **Identity (%)** | **Access Number** |
| FORCN047 | CB 01 | Santo Antônio | *Bacillus cereus* | 100.00 | [NR_074540.1](https://www.ncbi.nlm.nih.gov/nucleotide/NR_074540.1?report=genbank&log$=nucltop&blast_rank=1&RID=UJD3DR2Z014) |
| FORCN048 | CB 05 | Santo Antônio | *Bacillus cereus* | 100.00 | [NR_074540.1](https://www.ncbi.nlm.nih.gov/nucleotide/NR_074540.1?report=genbank&log$=nucltop&blast_rank=1&RID=9UGSSZ8F014) |
| FORCN049 | CB 08 | Santo Antônio | *Bacillus cereus* | 100.00 | [NR_074540.1](https://www.ncbi.nlm.nih.gov/nucleotide/NR_074540.1?report=genbank&log$=nucltop&blast_rank=1&RID=9UH7N2D9014) |
| FORCN050 | CB 09 | Triunfo Sample 1 | *Bacillus australimaris* | 99.93 | [NR_148787.1](https://www.ncbi.nlm.nih.gov/nucleotide/NR_148787.1?report=genbank&log$=nucltop&blast_rank=1&RID=9UH5889F015) |
| FORCN051 | CB 12 | Triunfo Sample 1 | *Bacillus australimaris* | 99.93 | [NR_148787.1](https://www.ncbi.nlm.nih.gov/nucleotide/NR_148787.1?report=genbank&log$=nucltop&blast_rank=1&RID=9NA1XZE1014) |
| FORCN052 | CB 15 | Triunfo Sample 1 | *Bacillus safensis* | 99.93 | [NR_041794.1](https://www.ncbi.nlm.nih.gov/nucleotide/NR_041794.1?report=genbank&log$=nucltop&blast_rank=1&RID=9N9X57VN015) |
| FORCN053 | CB 16 | Triunfo Sample 1 | *Bacillus safensis* | 99.86 | [NR_041794.1](https://www.ncbi.nlm.nih.gov/nucleotide/NR_041794.1?report=genbank&log$=nucltop&blast_rank=1&RID=UJD67PBH014) |
| FORCN054 | CB 17 | Triunfo Sample 1 | *Bacillus safensis* | 99.84 | [NR_113945.1](https://www.ncbi.nlm.nih.gov/nucleotide/NR_113945.1?report=genbank&log$=nucltop&blast_rank=1&RID=9UMN9XP801R) |
| FORCN055 | CB 18 | Triunfo Sample 1 | *Bacillus safensis* | 100.00 | [NR_041794.1](https://www.ncbi.nlm.nih.gov/nucleotide/NR_041794.1?report=genbank&log$=nucltop&blast_rank=1&RID=9UG5NA44014) |
| FORCN056 | CB 19 | Triunfo Sample 1 | *Bacillus australimaris* | 99.78 | [NR_148787.1](https://www.ncbi.nlm.nih.gov/nucleotide/NR_148787.1?report=genbank&log$=nucltop&blast_rank=1&RID=9UMM088701R) |
| FORCN057 | CB 20 | Triunfo Sample 1 | *Bacillus australimaris* | 99.64 | [NR_148787.1](https://www.ncbi.nlm.nih.gov/nucleotide/NR_148787.1?report=genbank&log$=nucltop&blast_rank=1&RID=9UZE1SBZ015) |
| FORCN058 | CB 24 | Triunfo Sample 1 | *Bacillus safensis* | 99.86 | [NR_113945.1](https://www.ncbi.nlm.nih.gov/nucleotide/NR_113945.1?report=genbank&log$=nucltop&blast_rank=1&RID=9UYAZY9W015) |
| FORCN059 | CB 32 | Triunfo Sample 2 | *Bacillus safensis* | 99.71 | [NR_113945.1](https://www.ncbi.nlm.nih.gov/nucleotide/NR_113945.1?report=genbank&log$=nucltop&blast_rank=1&RID=9UYH62G7015) |
| FORCN060 | CB 35 | Triunfo Sample 2 | *Paenibacillus illinoisensis* | 99.50 | [NR_113828.1](https://www.ncbi.nlm.nih.gov/nucleotide/NR_113828.1?report=genbank&log$=nucltop&blast_rank=1&RID=9UYDDE60014) |
| FORCN061 | CB 36 | Triunfo Sample 2 | *Bacillus safensis* | 99.86 | [NR_113945.1](https://www.ncbi.nlm.nih.gov/nucleotide/NR_113945.1?report=genbank&log$=nucltop&blast_rank=1&RID=9UYH62G7015) |
| FORCN062 | CB 45 | Triunfo Sample 3 | *Bacillus australimaris* | 99.93 | [NR_148787.1](https://www.ncbi.nlm.nih.gov/nucleotide/NR_148787.1?report=genbank&log$=nucltop&blast_rank=1&RID=9UYNR9X5014) |
| FORCN063 | CB 51 | Triunfo Sample 4 | *Paenibacillus barcinonensis* | 99.64 | [NR_042272.1](https://www.ncbi.nlm.nih.gov/nucleotide/NR_042272.1?report=genbank&log$=nucltop&blast_rank=1&RID=9UYRVFJD014) |
| FORCN064 | CB 52 | Triunfo Sample 4 | *Bacillus safensis* | 99.85 | [NR_113945.1](https://www.ncbi.nlm.nih.gov/nucleotide/NR_113945.1?report=genbank&log$=nucltop&blast_rank=1&RID=9UYTNTYF014) |
| FORCN065 | CB 56 | Triunfo Sample 4 | *Bacillus safensis* | 99.85 | [NR_113945.1](https://www.ncbi.nlm.nih.gov/nucleotide/NR_113945.1?report=genbank&log$=nucltop&blast_rank=1&RID=9UYTNTYF014) |
| FORCN223* | CB02 | Santo Antônio | *Bacillus safensis* | 100.00 | [NR_113945.1](https://www.ncbi.nlm.nih.gov/nucleotide/NR_113945.1?report=genbank&log$=nucltop&blast_rank=1&RID=UWA2NHJS014) |
| FORCN224* | CB03 | Santo Antônio | *Bacillus cereus* | 100.00 | [NR_074540.1](https://www.ncbi.nlm.nih.gov/nucleotide/NR_074540.1?report=genbank&log$=nucltop&blast_rank=1&RID=UWA54ACE015) |
| FORCN225* | CB04 | Santo Antônio | *Paenibacillus silvae* | 99.35 | [NR_156836.1](https://www.ncbi.nlm.nih.gov/nucleotide/NR_156836.1?report=genbank&log$=nucltop&blast_rank=1&RID=UWA6EM3N014) |
| FORCN226* | CB07 | Santo Antônio | *Bacillus australimaris* | 99.79 | [NR_148787.1](https://www.ncbi.nlm.nih.gov/nucleotide/NR_148787.1?report=genbank&log$=nucltop&blast_rank=1&RID=UWA7WSP7015) |
| FORCN227* | CB10 | Triunfo Sample 1 | *Lysinibacillus macroides* | 99.59 | [NR_114920.1](https://www.ncbi.nlm.nih.gov/nucleotide/NR_114920.1?report=genbank&log$=nucltop&blast_rank=1&RID=UWA9C34J014) |
| FORCN228* | CB11 | Triunfo Sample 1 | *Bacillus australimaris* | 99.79 | [NR_148787.1](https://www.ncbi.nlm.nih.gov/nucleotide/NR_148787.1?report=genbank&log$=nucltop&blast_rank=1&RID=UWABT0NW015) |
| FORCN229* | CB13 | Triunfo Sample 1 | *Lysinibacillus fusiformis* | 99.79 | [NR_112569.1](https://www.ncbi.nlm.nih.gov/nucleotide/NR_112569.1?report=genbank&log$=nucltop&blast_rank=1&RID=UWCFRYSE015) |
| FORCN230* | CB14 | Triunfo Sample 1 | *Lysinibacillus fusiformis* | 99.72 | [NR_112628.1](https://www.ncbi.nlm.nih.gov/nucleotide/NR_112628.1?report=genbank&log$=nucltop&blast_rank=1&RID=UWCHA63B014) |
| FORCN231* | CB22 | Triunfo Sample 2 | *Bacillus australimaris* | 99.79 | [NR_148787.1](https://www.ncbi.nlm.nih.gov/nucleotide/NR_148787.1?report=genbank&log$=nucltop&blast_rank=1&RID=UWADFU2N014) |
| FORCN232* | CB 23 | Triunfo Sample 3 | *Lysinibacillus fusiformis* | 99.93 | [NR_112569.1](https://www.ncbi.nlm.nih.gov/nucleotide/NR_112569.1?report=genbank&log$=nucltop&blast_rank=1&RID=UWCMPMVD015) |
| FORCN233* | CB25 | Triunfo Sample 4 | *Bacillus australimaris* | 99.72 | [NR_148787.1](https://www.ncbi.nlm.nih.gov/nucleotide/NR_148787.1?report=genbank&log$=nucltop&blast_rank=1&RID=UWAFRWTA015) |
| FORCN234* | CB30 | Triunfo Sample 2 | *Bacillus australimaris* | 93.27 | [NR_148787.1](https://www.ncbi.nlm.nih.gov/nucleotide/NR_148787.1?report=genbank&log$=nucltop&blast_rank=1&RID=UWAH4JVY014) |
| FORCN235* | CB31 | Triunfo Sample 3 | *Paenibacillus panacisoli* | 99.57 | [NR_041381.1](https://www.ncbi.nlm.nih.gov/nucleotide/NR_041381.1?report=genbank&log$=nucltop&blast_rank=1&RID=UWAMC36J015) |
| FORCN236* | CB33 | Triunfo Sample 4 | *Paenibacillus barcinonensis* | 98.67 | [NR_042272.1](https://www.ncbi.nlm.nih.gov/nucleotide/NR_042272.1?report=genbank&log$=nucltop&blast_rank=1&RID=UWAP58R7014) |
| FORCN237* | CB34 | Triunfo Sample 5 | *Bacillus nealsonii* | 99.10 | [NR_044546.1](https://www.ncbi.nlm.nih.gov/nucleotide/NR_044546.1?report=genbank&log$=nucltop&blast_rank=1&RID=UWARUKN8014) |
| FORCN238* | CB37 | Triunfo Sample 6 | *Paenibacillus barcinonensis* | 98.95 | [NR_042272.1](https://www.ncbi.nlm.nih.gov/nucleotide/NR_042272.1?report=genbank&log$=nucltop&blast_rank=1&RID=UWAUSDZ8014) |
| FORCN239* | CB39 | Triunfo Sample 3 | *Bacillus safensis* | 100.00 | [NR_113945.1](https://www.ncbi.nlm.nih.gov/nucleotide/NR_113945.1?report=genbank&log$=nucltop&blast_rank=1&RID=UWAWBCSP015) |
| FORCN240* | CB40 | Triunfo Sample 4 | *Lysinibacillus fusiformis* | 99.42 | [NR_112569.1](https://www.ncbi.nlm.nih.gov/nucleotide/NR_112569.1?report=genbank&log$=nucltop&blast_rank=1&RID=UWCP9UGS015) |
| FORCN241* | CB41 | Triunfo Sample 5 | *Bacillus safensis* | 100.00 | [NR_113945.1](https://www.ncbi.nlm.nih.gov/nucleotide/NR_113945.1?report=genbank&log$=nucltop&blast_rank=1&RID=UWAXVABF014) |
| FORCN242* | CB42 | Triunfo Sample 6 | *Bacillus safensis* | 99.93 | [NR_113945.1](https://www.ncbi.nlm.nih.gov/nucleotide/NR_113945.1?report=genbank&log$=nucltop&blast_rank=1&RID=UWAZAM30014) |
| FORCN243* | CB43 | Triunfo Sample 7 | *Bacillus aryabhattai* | 99.86 | [NR_115953.1](https://www.ncbi.nlm.nih.gov/nucleotide/NR_115953.1?report=genbank&log$=nucltop&blast_rank=1&RID=UWB0NKBX015) |
| FORCN244* | CB44 | Triunfo Sample 8 | *Lysinibacillus fusiformis* | 99.93 | [NR_112569.1](https://www.ncbi.nlm.nih.gov/nucleotide/NR_112569.1?report=genbank&log$=nucltop&blast_rank=1&RID=UWCRU4U7015) |
| FORCN245* | CB46 | Triunfo Sample 4 | *Bacillus australimaris* | 99.79 | [NR_148787.1](https://www.ncbi.nlm.nih.gov/nucleotide/NR_148787.1?report=genbank&log$=nucltop&blast_rank=1&RID=UWB257N5014) |
| FORCN246* | CB47 | Triunfo Sample 5 | *Lysinibacillus fusiformis* | 99.79 | [NR_112569.1](https://www.ncbi.nlm.nih.gov/nucleotide/NR_112569.1?report=genbank&log$=nucltop&blast_rank=1&RID=UWCRU4U7015) |
| FORCN247* | CB49 | Triunfo Sample 6 | *Paenibacillus barcinonensis* | 98.82 | [NR_042272.1](https://www.ncbi.nlm.nih.gov/nucleotide/NR_042272.1?report=genbank&log$=nucltop&blast_rank=1&RID=UWB4HPBK015) |
| FORCN248* | CB50 | Triunfo Sample 7 | *Paenibacillus barcinonensis* | 98.89 | [NR_042272.1](https://www.ncbi.nlm.nih.gov/nucleotide/NR_042272.1?report=genbank&log$=nucltop&blast_rank=1&RID=UWBF2HC5014) |
| FORCN249* | CB53 | Triunfo Sample 8 | *Bacillus safensis* | 99.93 | [NR_113945.1](https://www.ncbi.nlm.nih.gov/nucleotide/NR_113945.1?report=genbank&log$=nucltop&blast_rank=1&RID=UWBGFXZZ014) |
| FORCN250* | CB54 | Triunfo Sample 9 | *Bacillus aerius* | 99.79 | [NR_118439.1](https://www.ncbi.nlm.nih.gov/nucleotide/NR_118439.1?report=genbank&log$=nucltop&blast_rank=1&RID=UWBJ54RY015) |
| FORCN251* | CB55 | Triunfo Sample 10 | *Paenibacillus barcinonensis* | 99.36 | [NR_042272.1](https://www.ncbi.nlm.nih.gov/nucleotide/NR_042272.1?report=genbank&log$=nucltop&blast_rank=1&RID=UWBMEE74015) |

*Isolates that show reddish color according to the Gram test.
